# Fables of scarcity in IP

**DOI:** 10.3389/frma.2022.974154

**Published:** 2023-03-07

**Authors:** Zahr K. Said

**Affiliations:** University of Washington, School of Law, Seattle, WA, United States

**Keywords:** NFTs, copyright, artificial scarcity, intellectual property, aesthetics, narrative

## Abstract

In this chapter, I use methods drawn from literary analysis to bear on artificial scarcity and explore how literary and legal storytelling engages in scarcity mongering. I find three particular narrative strategies calculated to compel a conclusion in favor of propertization: the spectacle of need, the diversionary tactic, and the rallying cry. First, I unpack the spectacle of need and its diversionary aspects through several literary accounts of scarcity and starvation. I juxtapose Franz Kafka's “A Hunger Artist,” a story explicitly centered on a wasting body, with J.M. Coetzee's The Life and Times of Michael K. Second, to explore how scarcity fables offer diversionary tactics that redirect attention away from actual scarcity, I consider NFTs, or non-fungible tokens. NFTs reflect the arbitrary value scarcity can produce, especially when artificially generated. Yet NFTs offer a spectacle of need that distracts from actual scarcity, riding a wave of expansionist property logic that suggests that more ownership is the answer. Third, to consider the scarcity fable's propertarian rallying cry, I offer an extended close reading of a copyright dispute, Leonard v. Stemtech, involving a pair of microscopic stem cell photographs deemed so scarce they were valued at 100 times their past licensing history. Leonard illustrates how a scarcity fable may look in the context of intellectual property (“IP”). The nature of this chapter is necessarily conceptual and speculative, designed to raise questions rather than attempting conclusively to answer them. Through juxtaposition of literary accounts and one legal case study, fables of scarcity emerge as a genre whose very appearance in certain contexts ought to give scholars and policymakers pause. In copyright litigation, in which expansionist property narratives may be especially harmful to the public domain and subsequent creators, scarcity fables may be made to provide apparent support for potentially dangerous changes. Identifying scarcity fables as such when they appear in copyright cases could trigger review of the asserted scarcity and a more searching inquiry into whether the proposed solution could worsen actual scarcity.

## Introduction

Scarcity has long been theorized in different domains. Economists consider it in terms of supply and demand; psychologists understand it as a function of needs and wishes; sociologists map it on to hierarchies of taste and culture. More recently, digital marketers have fashioned myriad techniques to exploit it to their—and occasionally our—advantage^1^. There is nothing new, therefore, about positioning highly desirable things as scarce, regardless of whether they are empirically so as a function of resources or absolute value. Nor is there anything especially novel about the hunger to locate value as a function of rarity in a “post-scarcity” era in which the digital makes infinite duplication possible and thus destabilizes traditional valuation based on exclusivity, authenticity or presence^2^.

Some have argued that we are in a “post-scarcity” moment and sought to project the implications of a world in which technologies may “end scarcity as we know it.”^3^ In the digital plenitude of our post-scarcity moment, IP rights may arise “to artificially replicate scarcity where it would not otherwise exist.”^4^ This narrative captures the constructedness of both the claims about scarcity and the purported solution: property rights. An *actual* economics of scarcity exists, of course, and several important contributions to this volume—by Olufunmiyalo Arewa and Stephanie Bair, among others—offer important insights as they mine the implications of distributive disparities in connection with empirically scarce resources. However, here I engage mainly with the symbolic dimensions of scarcity as a construct. Such “artificial scarcity” consists of projections of need and desire that do not necessarily map with any accuracy onto an inventory of existing resources. Building on the work of Carol Rose, I adopt an understanding of scarcity as constructed, driven not necessarily by “natural disasters” but “simply an increase in humans' interest in the resource.”^5^ In Rose's memorable phrasing: “Nobody bothers to create property for some resource that lies around in abundance.”^6^ Indeed, through erecting property lines and creating exclusive rights, any resource can be made scarce^7^.

Under such an understanding of scarcity, we are likely never to reach “post-scarcity,” or a state of abundance, actually or conceptually. Artificial scarcity is not a necessary condition but a constructed one; it is of course possible for a civilization to make different sociopolitical, economic or cultural choices regarding its allocation of resources. Yet in our world, artificial scarcity is more the rule than the exception. In order to sustain the intentionally whetted appetite for exclusive ownership of rare and valuable things, we determine what counts as rare and valuable and then decide to continue fencing those things off to keep them that way. In service of such fencing, I posit, come what I call “fables of scarcity.”^8^

Drawing on Vivian Gornick's distinction between the “situation” and “the story” is helpful (see footnote 6). In a scarcity fable, the narrative action is grounded in the present problem (the “situation”) which presents a seemingly unresolvable set of challenges causing impoverishment, famine, thirst, infertility, and the like. This need (compulsion, desire or even fervent wish) creates a nihilistic horizon, an inevitably increasing lack and dystopian future characterized by asymptotic scarcity and relentless suffering. Bleak futurescapes shaped by the situational need generate narrative tension for “the story” (drawing again on Gornick's terms). The story set off by the situation, in other words, is a tragedy or disaster that will unfold unless something intervenes. It is this story of predicted suffering to which the reader or recipient responds, the emotional pitch to intervene… or else. In turn, the story builds momentum through a form of “scarcity mongering,” drumming up the reader's desire for resolution in the form of quenching the lack established at the story's outset.

Fables of scarcity commingle and confuse true need with its less urgent forms like desire and compulsion. Accordingly, subjective allegations of need get bound up with equally subjective claims of value, rarity and uniqueness and deployed in even more subjective descriptions of resources as dwindling or under attack. However, this fabular accounting may not map accurately onto an existing inventory; the point instead is its very deployment. The intense deprivation serving as the story's impetus justifies a persuasive call for action; *something* needs to happen because of the dire need. It also provides cover. Indeed, the very structure of the scarcity fable distorts the process of accurately inventorying resources since a signal aspect of this genre is diversion of attention away from actual scarcity and onto the fantasies of abundance called into necessity by the spectacular (but subjective) lack.

Structurally speaking, fables of scarcity are formulaic narratives. The situation opens with a gap to be filled or a severe problem to be solved. I call this the “spectacle of need” because it sets up potential diversion and launches a kind of narrative legerdemain. The spectacle of need is no ordinary statement of need, but an entrancing, possibly sweeping presentation of deprivation that establishes the narrative conflict in propertarian terms and serves as a diversionary problem story that establishes a lack (or a painful longing) not necessarily tailored to an accurate inventory. Unlike an accurate accounting of resources or unemotional tallying of what remains for use, the situation establishing the need upfront is fabular. Like other stories that operate in the realm of the symbolic, the scarcity fable exists in an amplified, emotional register rather than an empirical one. Bedazzled by the spectacle, one ceases to look probingly at the resources in question. This first movement in the scarcity fable is thus distracting and distorting, even as it establishes narrative conflict.

The scarcity fable, like any other conflict-oriented story, next features a struggle. There will be some progress gained and lost as various efforts ultimately fail to improve the dire foundational situation meaningfully. These narrative vicissitudes are designed to maintain audience interest while merely prolonging the inevitable. Put another way, the spectacle of need leads to a struggle that feels, at least temporarily, futile. *The narrator has tried everything! Nothing has worked! Nothing will work!* This asserted futility paves the way for resolution and abundance or failure and the triumph of scarcity. The spectacle of need diverts attention from actual scarcity and sharpens the stakes for a propertarian “pitch” to come, a rallying cry for resolution in the form of ownership or exclusion.

Fables of scarcity may bifurcate into one of two common points of resolution. They may resolve the scarcity with abundance, culminating in some form of narratively engineered “more.” Alternatively, they may close off the possibility of resolution with a dystopian refusal of abundance. Plots that resolve happily may call to mind the ending of Shakespearean comedies, which tend to end in marriage (often, multiple marriages). Abundance can be restored, but its promise is contingent on some sort of action: a purchase; matrimony; newfound generativity (in the form of grandchildren or a seed that has borne fruit). In turn, the action promises *more*—more food, more technology, more property, more safety, more freedom, simply more of whatever is missing—and this ostentatious “more” delivers release from the suffering scarcity imposes. In plots that resist such closure, the unresolved spectacle of need leads to a barren, empty future; unremitting suffering; and ultimately, death.

In this chapter, I bring textual analysis to bear on artificial scarcity and explore how some literary and legal storytelling engage in scarcity mongering. This form of persuasive rhetoric reflects three particular narrative strategies that seem calculated to compel a conclusion in favor of propertization: the spectacle of need, the diversionary tactic, and the rallying cry.

First, I unpack the spectacle of need and its diversionary aspects by considering several literary accounts of scarcity. Franz Kafka's “A Hunger Artist,” is a story explicitly centered on a wasting body. Kafka's story casts scarcity in terms of a modernist aesthetic one critic has termed “the art of hunger.”^9^ This aestheticized hunger emerges as a modernist lament about the conditions of creating art under capitalism, with the artist's body registering market pressures. Later literary and aesthetic movements advance the art of hunger in service of different ideological messages, suggesting that fables of scarcity enable cultural and political critique of artistic production. The scarcity fable, as I define it, can be mobilized both as a critique of capitalist conditions or indeed, an unlikely paean for them. It also holds significant power as a political parable, as I discuss with respect to J.M. Coetzee's *The Life and Times of Michael K*, a work explicitly in dialogue with Kafka's story.

Second, to explore the ways in which scarcity fables offer diversionary tactics that redirect attention away from actual scarcity, I consider NFTs, or non-fungible tokens. NFTs are the latest iteration of a longstanding cultural fascination with the scarce, and they reflect the often rather arbitrary value scarcity can produce, especially when artificially generated. NFTs have been heralded as a technological solution to a technologically enabled problem: in a world now saturated with digital copying, unique embodiments of a work have grown scarce. Yet NFTs offer a spectacle of need that attracts attention and distracts from actual scarcity, riding a wave of expansionist property logic that suggests that more ownership is the answer.

Third, to consider the scarcity fable's propertarian rallying cry, I offer an extended close reading of a protracted copyright dispute. *Leonard v. Stemtech* involved a pair of microscopic stem cell photographs deemed so scarce they were valued at 100 times their past licensing history^10^. From the assiduous and skilled photographer's difficulty earning a living and the rarity of these two photographs to the proposed “solution” in the form of significantly multiplied damages, the case offers an example of a scarcity fable in the context of intellectual property (“IP”). *Leonard* illustrates the operation of the spectacle of need and highlights how these fables can build to a crescendo, a narrative rallying cry in favor of property.

A common thread underlying the chapter is the way in which structuring stories around artificial scarcity militates in favor of expanding property rights. Consequently, the first two parts of the paper build most of the scarcity fable's theoretical framework before the third part turns to a legal context in which the real-world stakes of such fabulism become clear.

## Artificial scarcity as impetus (the spectacle of need)

Franz Kafka's short story, “A Hunger Artist” (1822) offers a paradigmatic scarcity fable, a story that appears to center on one kind of deprivation when in fact masking another. “A Hunger Artist” imagines a performer whose gambit is to position himself in a public place and fast for 40 days^11^. His partner in this venture is “the impresario,” a front man who drums up audience interest in the show. Together, the two have traveled around Europe tapping into spectators' interest in this ritualized display of abnegation. In each location, on the fortieth day—which their informal market research has identified as the peak of audience interest—the hunger artist is brought out before the audience and forced to eat (HA, 246–247). He laments having to do so, certain that he could fast for longer, but the market imperative holds sway and each time, the hunger artist plays out the scene according to the impresario's directions (HA, 247, 249). Eventually, interest in the phenomenon of fasting wanes and audiences shrink (HA, 250). He and the impresario part ways. The hunger artist is forced to take up with a traveling circus and “perform” his fasting in a cage on a bed of straw, exhibited like an animal (HA, 251).

While with the circus, the hunger artist further recedes in importance and visibility. Soon he is forgotten altogether, even by the circus employees charged with daily updating a sign that boasts the number of days he has been fasting (HA, 252–253). Without the daily count that marks and structures the spectacle, “the artist simply fasted on and on, as he had once dreamed of doing” (HA, 254). His internal monolog registers the ease of extending the fast (“it was no trouble to him, just as he had always foretold”) yet whatever satisfaction that might once have generated seems fleeting (HA, 254). The artist discovers that since nobody is counting the days, “no one, not even the artist himself, knew what records he was already breaking, and his heart grew heavy” (HA, 254). A circus supervisor discovers with some surprise “this perfectly useful cage … standing there unused with dirty straw inside it” and asks an underling about it (HA, 254). At first nobody can answer but then an employee, “with the help of the table with the number on it, remembered the hunger artist. They pushed the straw around with a pole and found the hunger artist in there,” barely alive long enough for a condescending exchange of remarks in which the artist, in his dying words, purports to confess his motivations. (HA, 254–255)

The hunger artist's reasons for “performing” his fasting in this way are complex. He seeks attention; he knows no other skill; he wants people to understand that he can fast and indeed attempts to prove his honesty by singing, to offer as proof his empty mouth; he needs people to understand that he *must* fast, in other words, that he is bound by some inner compulsion to fast; finally, he confesses that he has never discovered a food that he enjoys or else he would have, like ordinary people, gorged on it: “If had found that, believe me, I would not have made a spectacle of myself and would have eaten to my heart's content, like you and everyone else”. (HA, 255). The text immediately undercuts the hunger artist's justification. “These were his last words, but in his dimming eyes remained the firm though no longer proud persuasion that he was still continuing to fast.” (HA, 255) Kafka reminds the reader of the hunger artist's compulsion which is “no longer” a source of pride but conveys something else, whether stubbornness or compulsion, that reflects an unmet need.

The short story can be read as a parable of individual loneliness and ascetism and a lesson about the failures of the capitalist economy, the offensiveness of callous transacting in the face of human suffering. Along those lines, Robin West and Richard Posner have sparred over their competing interpretations of the piece^12^. West finds in the work hints of the tension between true autonomy and a market that may, for the right price, induce behaviors or consent that cannot be morally or ethically justified (such as selling admission to the spectacle of one's own starvation)^13^. The story serves as a vehicle for her critique of Posner's law and economics. Posner's acerbic response rejects West's reading of the story: “A Hunger Artist” “may be about many things. But only superficially is it about hunger, poverty, the pitfalls of entrepreneurship, and the fickleness of consumers.”^14^ Posner's point is that the story's meanings should not be reduced, especially in service of what he views as West's instrumentalist critique, to a single-minded or literal view as though the marketplace were real and the hunger representative of actual hunger. In turn, West notes in her response to Posner's critique of her reading that many of Kafka's works “are unquestionably, as Posner tells us over and over, ‘about' religious authority, familial authority, Oedipal complexes, the overbrooding conscience, the neurosis of the sensitive soul's inner life.”^15^ Yet reading them as sources of multiple themes and for divergent meanings does not threaten to oversimply or reduce Kafka. Instead, the story, like much of his other work, exists on a symbolic plane and resists attempts to reduce it to singular meanings. Kafka critics tend to have converged on this view of the text as well, namely that its function as an allegory makes “A Hunger Artist” capable of bearing many meanings and incapable of settling on a single one^16^.

“A Hunger Artist” can also be read as a fable of scarcity, and specifically, artificial scarcity driven by an appetite not matched by existing resources. This scarcity fable opens with a spectacle of need, only the spectacular need in question is not the hunger artist's need for food but his drive for something else. Perhaps he needs his performance to find a market; or perhaps he desires an audience to attest to his compulsion to fast. His is not a hunger strike, an obvious political statement of autonomy or resistance^17^. The narrative conflict arises as a question bearing a sense of tragic inevitability: will something not arrest his slide into death as he continues to choose to perform his own starvation? Will he not find his place, his audience, or whatever might curb his yearning to be noticed and believed? If nothing changes, the hunger artist will recede into nothingness, the victim as much of his fasting as of the audience's indifference. The story ends on a note of unremitting scarcity for the hunger artist, a resounding refusal to gratify his need for an audience or witness and his death by starvation. He is replaced by a contrasting figure of intense abundance, a panther who occupies the cage exuding muscular energy, “his noble body, furnished almost to the bursting point with all that it needed,” and seeming “to carry freedom around with it,” “the joy of life stream[ing] with … ardent passion from his throat” (HA, 255). Kafka permits a brief glimpse of a future free of dire need (note the animal's characterization as “furnished… with all that it needed”). Yet Kafka permits this possibility of abundance only for an exotic circus animal whose non-human characteristics are reinforced by the fact that “He seemed not even to miss his freedom” (HA, 255). For the human artist, such a future is unimaginable.

Kafka's scarcity fable can be productively juxtaposed with another story that centers starvation, if in an utterly different context. J. M. Coetzee's *The Life and Times of Michael K* (1983), tells the story of a grown man of color with ostensibly low functioning skills and a facial disfigurement in the form of a hare lip that prevents his mouth from closing fully^18^. Set in South Africa during a non-specified period of imagined civil war in the late 1970s or 1980s, this dystopian novel follows Michael K during this tumultuous time in his life and in the country. The fictionalization of a war operates, like many other elements of the work, on an allegorical plane.

K is not close to his mother, Anna K, who abandoned him to a city orphanage for much of his youth, partly due to his facial disfigurement and its social impact on both of them (LT, 4). Yet when he learns that she is dying, he finds deeper purpose in agreeing to care for her (LT, 5,7). Hearing that layoffs loom over K's job as a gardener tending to the city of Cape Town's parks, Anna asks K to take her to the countryside where she was born (LT, 7–8). K agrees and ceases to report to his job starting the next day (LT, 9). This employment is the first of many losses that will dog him throughout the story.

K and his mother seek formal permission and traveling papers to leave the city but are refused (LT, 9). Facing difficult circumstances no matter whether they remain or attempt to travel illegally, they deliberate for a few days. Cape Town falls under siege which results in their eviction from the home in which they are temporarily residing. They harden their resolve to leave, make an attempt to escape and fail (LT, 18–22). A second attempt to escape launches more successfully. K, transporting his ailing mother by wheelbarrow, sets off on a journey the reader understands to be doomed from the start: his mother knows only the name of her village and the way the homestead and garden looked—she has no address (LT, 27). Moreover, they are traveling illegally during armed conflict and in bad weather as people of color without privileges, papers, or power and his mother is unwell even at the start of their travel (LT, 23). The difficulties are heightened when, en route, his mother dies and K is left alone without employment or prospects, soon robbed of his wheelbarrow and most of his remaining possessions (LT, 30, 34).

K experiences a long sequence of challenges including forced labor, imprisonment and risky escapes, followed by multiple phases of prolonged starvation. This “struggle” phase introduces K's uncanny green thumb, humility and determination even as he is dispossessed of his valuables. K's gardening skill leads to brief, painstakingly earned successes as he turns seeds into pumpkins and feeds himself off the land on which he is squatting (LT, 59). Yet these horticultural successes are interrupted by diverse external forces both serious and absurd (LT, 65, 101, 111). The plantings are lost or destroyed, whereupon he begins again; again his work is undone and the plants and provisions lost. He leaves the land and is imprisoned but escapes and returns to it, with similar results. The novel prefigures and naturalizes K's lifelong struggles with hunger with its opening image of K as a young baby born with a “mouth that would not close” which caused his mother revulsion as he struggled either to nurse or to accept a bottle (LT, 3). K seems unable to escape famine and resolved to his own destiny, displaying indifference, at times, to the extent of his own hunger.

A sense of futility settles over the story as K several times descends into starvation so serious it is hallucinatory (LT, 117, 118, 129). He ultimately requires extended hospital care to reverse his malnutrition. The narrative flits in and out of realism as it conveys the sense of mystery and confusion a patient in such circumstances might experience, relaying the events through the perspective of a nameless medical officer who disdains but, in the psychosocial alienation of wartime, in some sense also comes to depend on K.

The novel refuses any gesture of restorative abundance until the end when a spare vision of possibility, if not plenty, emerges. K escapes the hospital without ever having willingly eaten or yielded the information sought from him. He encounters strangers who feed, intoxicate and seduce him, catalyzing in K a newfound awareness of the need to be independent of charity despite the pressures of unemployment, homelessness, famine and growing drought. In a narrative voice strikingly different from earlier points in the novel, K imagines gaining sufficient capacity to offer charity to others. The key, as he muses to himself, is patiently waiting.

[I]f there was one thing I discovered in the country, it was that there is time enough for everything. (Is that the moral of it all, he thought, the moral of the whole story: that there is time enough for everything? Is that how morals come, unbidden, in the course of events, when you least expect them? (LT, 183)

K's capacity to survive in the country appears to have sharpened his agricultural skills but also delivered perspective. Yet what appears at first to be hard-won wisdom comes to look more like delusion or magical thinking. K imagines “a little old man” who appeals to him for help finding water in drought-ridden Cape Town. K, casting himself as hero in this invented scenario, loops a long string over a teaspoon and lowers it down into the ground as though tapping a well. Despite the implausibility of this strategy, the novel ends on a quasi-magical note: “when he brought it up there would be water in the bowl of the spoon; and in that way, he would say, one can live” (LT, 184).

The contexts of Kafka's and Coetzee's stories are different, to be sure, as are the conflicts (or incentives) driving the characters' starvation. Still, the juxtaposition of the two works is not infelicitous: the K in the story's title is thought to be a nod to Kafka^19^ (an author to whom Coetzee admits his indebtedness^20^); the administrative hassles throughout Coetzee's novel are nothing if not Kafkaesque; and Coetzee's journal reflects an entry linking the novel to Kafka's short story thematically^21^. Both stories are also parables haunted by a similar mood of desolation, even or especially when an individual is among others in a crowd. Both feature a character on the verge of starving to death and ultimately forgotten^22^. Finally, both aestheticize hunger “within the art of hunger [literary] tradition”^23^ and cast the refusal to eat as a form of symbolic resistance^24^.

The hunger artist's craving exemplifies the spectacle of need, which is not an accurate accounting of needs and resources but rather a displacement, a diversion away from the actually urgent needs of his body. In failing to find either a willing audience or a fresh purpose, the hunger artist overlooks the actually dwindling resource: his own body. Nutritional scarcity is never, to be clear, remotely a pretext for the hunger artist's fasting; that's what makes his fasting art (or at least spectacle) as opposed to inevitable suffering to which there happen to be powerless bystanders bearing witness. On the contrary, the hunger artist laments a different kind of scarcity. As time goes on, he identifies what today we might call attention scarcity^25^. He starves to death, his struggle to reconcile the oversupply of his “talent” with the disappearing demand for it.

Michael K's rejection of food differs from that of the hunger artist. His refusal to eat while imprisoned signals the germination of a new sense of autonomy, of freedom, in other words, whereas the hunger artist's refusal appears as a form of compulsion, even implicating pride or ego^26^. Yet when faced with actual hunger, and actually scarce resources, the characters behave differently. The hunger artist's choice to go hungry, to martyr himself for his “art,” looks positively decadent in a world in which Michael K spends half a day lying on the ground with his face poised over an ant nest, “picking out the larvae one by one with a grass stalk and putting them in his mouth” (LT, 146)^27^. One scholar has observed that the aestheticization of hunger (in Kafka and Coetzee) represents the privileged posture of representing hunger vicariously. He writes that “In South Africa, the art of hunger therefore becomes fraught in a new and newly politicized way: as a literary tradition it belongs to the European lineage that is the preserve of white authors like Coetzee; as an experience—as a form of hunger—it is the province of apartheid's non-white population.”^28^

This insight underscores that the *kinds* of scarcity and need in the two works are different, even if the outer biological phenomena, the barely-there skeleton, the system shutting down, are superficially the same. Ordinarily the discernible scarcity associated with a starving body would be attributed to a lack of food (and in K's case, a lack of freedom to work or farm and the resource-starved conditions of war). But Kafka's hunger artist's death is not caused by scarcity of food *per se* but by an inner compulsion to resist food even though it is available to him. In this way, he is like Bartleby the Scrivener, the eponymous main character in Herman Melville's 1853 short story, a character Coetzee also had in mind in writing about Michael K^29^.

Bartleby is employed as a “scrivener” at a law firm doing “an extraordinary quantity of writing” (BS, 114). The story casts his productivity in terms of an unusual hunger: “As if long famishing for something to copy, he seemed to gorge himself on [his employer's] documents. There was no pause for digestion” (BS, 114). Bartleby's appetite for work could have been cause for celebration but his employer, the narrator, conveys something unhealthy about it from the start: Bartleby “wrote on silently, palely, mechanically,” and without pausing for “digestion.” (BS, 114)

On the third day of his employment when asked to review a document, Bartleby responds “I would prefer not to” (BS, 115). At first, he continues to work as a copyist but politely declines direct requests. Despite the narrator's consternation, this phrase (and posture of inaction and silence) become Bartleby's signature reply, as his conduct continues to become more unusual and antisocial. The narrator discovers that Bartleby has begun secretly living in the office (BS, 122). So long as Bartleby maintains productivity, however, the narrator is inclined to tolerate his employee's odd mannerisms. He often expresses sympathy for Bartleby and maintains a view of the latter's innocence. Bartleby's workload continues to dwindle until 1 day when he spends his working hours staring out a window that looks onto a brick wall (BS, 127). When queried about his refusal to work, he announces “I have given up copying,” (BS 128)^30^. Bartleby becomes an increasingly significant obstacle for the narrator, who resolves to fire him but somewhat inexplicably fails to manage to do so (BS, 134, 135). Feeling powerless to adopt any other course of action, the narrator vacates his own law offices and sets up his business elsewhere to avoid having to confront Bartleby (BS, 135–136). Eventually, Bartleby is arrested for vagrancy and—despite the narrator's interventions—dies in prison, reportedly refusing to eat. The narrator relays seeing “the wasted Bartleby” one last time, “huddled at the base of the wall” in his cell, “his knees drawn up and lying on his side.” Bartleby is motionless, “his dim eyes open” though “otherwise he seemed profoundly sleeping” (BS, 142).

Bartleby's refusal to act is inexplicable, something not grounded in any narrative justification and highly unusual in the context of nineteenth-century fiction. It is possible retrospectively to pathologize Bartleby, viewing him through contemporary psychology and diagnosing him in various ways as modern critics have done. For instance, his behavior now seems symptomatic of depression and perhaps anorexia^31^. Yet Melville's story itself refuses any tidy explanation, preferring instead to emphasize the unavailability of Bartleby's motivations as “unascertainable.”^32^

Similarly, the hunger artist is a character whose stubbornness or compulsion finds no full justification in the text. Both he and Bartleby exist on an allegorical plane in which interpretive finality or fixity is withheld. Both stories are not merely capable of multiple readings but indeed incapable of a singular one. Part of their powerful appeal comes in holding mysteries that cannot be answered in the narrative terms of the texts; that is, both of these stories refuse narrative closure and insist on their own allegorization.

Whatever its many levels of signification, “A Hunger Artist” is also a scarcity fable. In the inner logic of the story, the true scarcity is framed as the collapsing market for what the hunger artist has, or knows how to do; Kafka reveals “a deep anxiety about the relationship of art and the market” and undermines “the possibility of autonomous art in a commercialized context”^33^ What the hunger artist knows how to do is fast for extended periods of time, practically punching in on the clock and devoting himself to his profession with a work ethic to be celebrated under bourgeois ideologies of labor and selfhood^34^. He is desperate to fast, and arguably to be the model worker as he does so; he is less political martyr than laborer seeking validation for his value within “the system” as a changing market has left him behind^35^. Read in this way, “A Hunger Artist” is also a parable of collapsing business models that would rather die—ostensibly martyred on account of some form of market scarcity—than reinvent themselves. Which brings me to NFTs.

## Artificial scarcity as diversion (masking actual scarcity)

NFTs have been hyped as revolutionary, having surged into public view in 2017 with something like a technoaesthetic manifesto, a pret-a-porter philosophy of art that champions distributed-ledger technologies and seems to promise the democratization of both art and technology^36^. They have also been offered as a nifty solution to a set of challenges associated with creating and transacting in works of digital art. However, what appears to drive the NFT hype is not NFTs' capacity for verification and recordkeeping but rather their immediacy and allure of purported exclusivity. In some sense, NFTs create a semblance of presence, forging a connection with the asset in connection with which the token is being minted. Indeed, NFTs seem calculated to “solve” for a more ineffable problem, the impossibility of uniqueness in our ubiquitously digital, everything-is-replicable moment. As one industry insider explained, “Once something is copied and replicated for free, the value drops and the prospect of a market disappears. For things^37^ to be of value they need to have scarcity. Blockchain helps solve this for digital artists by introducing the idea of ‘digital scarcity': issuing a limited number of copies and tying them back to unique blocks proving ownership. Scarcity is thus the condition, the diagnosis and the question; NFTs are the cure and the answer. According to their champions, their technological affordances permit NFTs to restore a form of abundance to a world starved for the unique or non-replicable^38^. This ironically non-ironic account of NFTs tells a particular story about scarcity that it is worth unpacking in terms of the notion of scarcity fables. NFTs seem to hold somewhat internally contradictory promise: in the eyes of proponents, they drive sales in digital art through creating “digital scarcity” yet–paradoxically—they will also democratize fine art (ostensibly by disintermediating various creative markets in digital works and supporting both fan and creator communities).

Given widespread confusion in popular discourse and journalism about NFTs, let us revisit what an NFT actually *is*. An NFT is a non-fungible (i.e., unique, non-replicable) token associated with some asset. NFTs resemble Bitcoin in that transactions over both are recorded on the blockchain. But they differ in that Bitcoin is fungible—each unit is not unique relative to each other unit—whereas NFTs are unique tokens associated with an asset^39^. It is the *token* that is unique, to be clear; not necessarily the asset. That asset could be an original work of art, a licensed copy, a fake, or any number of other types of “thing” such as a tweet,^40^ an article,^41^ a picture of a newspaper article,^42^ a meme,^43^ a clip of a basketball game,^44^ a series of musical videos,^45^ an audio sex tape,^46^ and so on. There are different kinds of NFTs, but the most common kind consists of “a metadata file that contains information that has been encoded with a digital version of the work that is being tokenized.”^47^ A “tokenID” is paired with “a blockchain address” and together these two elements make the token unique^48^. Most NFTs also include a link that refers to where the original work is stored online, which underscores that the NFT is *not* the same thing as the asset with which it is associated^49^. They also link back to the creator's wallet so as to indicate minting provenance (see text footnote 47).

Because much of the hype around NFTs conflates the things to which the token refers with the tokens themselves, I find it helpful to frame NFTs in terms of a metaphor^50^. The NFT's token—typically comprising the tokenID and blockchain address plus some additional information as noted—is somewhat like a luggage tag. To be sure, it is a unique and non-fakeable luggage tag, that can identify any person's particular piece of luggage. There is nothing necessarily authenticating about NFTs with respect to the asset in connection with which the NFT was minted. A unique and non-fakeable luggage tag could be generated either in connection with a fake Louis Vuitton bag purchased by a purchaser who knows very well they are purchasing a fake or in connection with a sneaky counterfeit a buyer incorrectly believes to be authentic. The luggage tag tells buyers nothing more than that this was a correct match for the one particular piece of luggage in question, a piece of luggage which might not be unique in manufacture in any way, but whose contents are particularized to its tag holder (the owner of the NFT). The NFT itself cannot verify whether the asset (the luggage) is real or fake ab initio; it can only verify that the asset associated with this token is the same asset that was originally associated with it.

Leaning even more heavily on the analogy, if a traveler checked in a piece of luggage in Dallas and claimed it in Atlanta, that luggage tag verifies merely that it is the same piece before and after the journey; it permits accurate retrieval by the correct owner. But that is all that it does. If, once at your destination, you sell your luggage to another traveler, the luggage tag will be updated to reflect that you have done so, and from that point forward, the tag will identify another owner until any subsequent transfers. Here the analogy highlights how NFTs foster confusion. Claiming the luggage by using this luggage tag does not guarantee anything about its authenticity; it only makes an ownership match. Moreover, owning the luggage tag does not necessarily provide any special rights over the luggage contents. Extending the conceit a little further, if the luggage held lawfully purchased CDs and DVDs containing audiovisual works, the rights to claim ownership of the luggage tag and to store the contents of the luggage would not convey the right to duplicate the music or screen the films publicly^51^. (Private uses are of course within the scope of possessing the CDs and DVDs, and fair uses do not even depend on ownership at all). Absent further contracting to ensure the transfer of use rights, NFT ownership is not much more than collecting luggage tags so you can brag about the contents^52^.

It is easy to lampoon NFTs as deceptively insubstantial collectibles, “Like beanie babies without the beans,”^53^ or worse, an outright con, “a new kind of magic bean to sell for actual money, and pretend they're not … magic beans.”^54^ Yet sophisticated purchasers—and perhaps collectors generally—likely know what they are getting^55^. Even as mere luggage tags, NFTs clearly could have some value, and hold possible promise for their capacities for online identification and verification; market disintermediation; community-building; and creative innovation.

First, like other distributed ledger technologies, NFTs possess the capacity for online identification and verification. They can create, maintain and confirm records of historical transactions^56^. NFTs have been offered as solutions to several different kinds of problems pertaining to sales and ownership online. For instance, it has been claimed that there used to be “no way to separate the “owner” of a digital artwork from someone who just saved a copy to their desktop” and this uncertainty promoted unlawfulness and stymied business: “[m]arkets can't operate without clear property rights” (see text footnote 56). NFTs can permit verification of ownership records and transactions and thus make it harder to unlawful use or sell items owned by others^57^. NFTs may also induce trust through their capacities to safeguard transactions online by allowing a purchaser to verify the authenticity of the item to be purchased^58^. NFTs are thus thought capable of promoting authenticity, reducing forgery, and minimizing piracy online (see text footnote 38)^59^. In sum, NFTs are imagined as a pragmatic solution to the multifarious challenges posed by online transactions in digital assets, given the fungibility of digital copies (see text footnote 38, 59).

NFTs have also been promoted as a means of eliminating the middleman in transactions that remunerate artists, thus lending NFTs a populist appeal for those who would like to support their favorite artists more directly^60^. In some accounts, NFTs hold the key to democratizing the world of art and heralding an unprecedented middle class full of potentiality for creators^61^. In one refreshing contrarian take, NFTs offer a promising means of doing away with copyright and offering artists and purchasers more of what they actually want^62^. In theory, NFTs cut out the middleman and thus enable artists to transact directly with audiences and interested buyers^63^. NFTs could create a form of democratized, decentralized patronage that disrupts existing business models (see text footnote 38). However, it is unclear how, in minimizing traditional intermediaries, the NFT market has not replaced them with newer intermediaries in the form of NFT platforms^64^. If NFTs could live up to their promise as a means of remunerating creators and cutting out the middle-man, they would be socially valuable for many on those grounds alone^65^.

Generally, NFTs are also thought to foster greater connection among fans and with creators. Proponents further argue that NFTs can be used in ways that build community and foster innovation because they can be “endow[ed]… with features that enable them to expand their purpose over time, or even to provide direct utility to their holders. … In this sense, NFTs can function like membership cards or tickets, providing access to events, exclusive merchandise, and special discounts — as well as serving as digital keys to online spaces where holders can engage with each other.”^66^ Finally, NFTs could also represent—as some artists believe of some forms of NFTs—an innovative frontier of creativity in direct lineage with Andy Warhol^67^.

Joshua Fairfield offers a compelling account in this volume, discussing the rise of NFTs as one response to the perceived risks associated with potentially endless copying of digital goods, a response intended to capitalize on technological tools to create “rivalry, scarcity and uniqueness.”^68^ As Fairfield notes, NFTs are “database entries, written to a smart contract,” and things that “often do not represent value merely by themselves.”^69^ Sometimes these tokens are merely pointers to some valuable thing hosted somewhere else; other times they may incorporate the valuable thing through “a hash of the entire [work], a number generated by running all of the pixels …. through a mathematical function that creates a unique math string of limited length.”^70^ In many instances, the buyer may own the unique hash of the work without specified use rights that govern the things non-collector humans actually care about doing with that work, i.e., playing it as a video rather than storing it somewhere as a converted string of numbers. In other words, the purchaser of an NFT may be owning nothing more than a certified-mail-version of a url—a unique link to somewhere, often public, where others may also view that given work—and where they have no rights other than claiming their ownership in the certified mail receipt. Alfred Steiner points out that this single characteristic may be culturally and epistemologically valuable in and of itself: “NFTs will also make art history a bit easier by providing definitive proof of who did what when. If nothing else, NFTs are the ne plus ultra of the timestamp—the Twenty-first Century equivalent of posting a sealed letter to oneself.”^71^ Notwithstanding this important point, the buzz around NFTs would seem to promise more. Despite their possible benefits to brand owners, token holders, creators and fans, NFTs generate significant costs of multiple kinds and some of its proponents downplay or ignore the extent of these costs.

First, considerable *uncertainty costs* attach to NFTs. These are standard risks associated with new legal modes and business practices: what sorts of licenses are required to convey (or limit) NFTs and how will traditional terms be construed in the context of smart contracts for tokenizing digital assets? In short, the definition of “ownership” of NFTs is uncertain and “the technological answer may not always conform to the legal answer.”^72^ The rhetoric of “digital ownership” is unclear and sometimes downright obfuscatory; one critic opines that “The more detail you ask for what actual usable rights this “ownership” conveys, the vaguer the claims will get” (see text footnote 54). This lack of certainty is not necessarily all bad. Fairfield describes the benefits flowing from NFTs' flexibility and modularity, for instance. That there are innovative possibilities for defining and enforcing ownership interests could be beneficial. He cautions, however, that the costs of such modularization grow with complexity and thus create corresponding costs and risks^73^.

These uncertainty costs also apply to exclusive rights in IP. What sorts of conduct counts as infringing with respect to minting NFTs?^74^,^75^ There is no existing regulatory mechanism to prevent unauthorized uses of IP in the minting of new tokens, and once an NFT is minted, removing it from the blockchain is apparently impossible. New lawsuits appearing over NFTs point to the potential stakes of such uncertainty^76^. Recently, members of Congress requested that the USPTO study the intersection of NFTs and IP rights, underscoring the potentially significant stakes of this ongoing uncertainty^77^. The issues around ownership and transfer of rights in assets associated with NFTs are complex and require an understanding of, inter alia, the derivative work right; fair use; limitations on rights such as the lawful owner's right of public display; and the intersections and distinctions between trademark and copyright law, which few members of the general public are likely to have^78^.

Second, and relatedly, there are *confusion costs*: many members of the ordinary public do not understand blockchain technologies, let alone this latest use case. Fewer still are likely to be able to navigate both the technological and legal implications of transacting in NFTs. This may confuse buyers who attach the wrong meanings to the “scarcity” associated with NFTs. As one industry insider has put it, “Essentially, NFTs create digital scarcity,” which creates value with respect to digital assets, whose supply is otherwise—at least theoretically—limitless^79^. Minting a unique token which requires resources on the blockchain to signify and guarantee its uniqueness is what generates value^80^. Yet this artificially generated scarcity can just as artificially disappear since a creator can mint as many tokens as they wish in connection with a work; nothing guarantees that subsequent minting will not dilute the value of the token, in other words^81^.

This is troubling, given that NFTs are touted as a means of providing unique value; one of the primary drivers behind NFT ownership is the notion that “owning” a rare item is an unusual opportunity and should be priced accordingly. If NFTs can be diluted in this way through post-sale issuance, what precisely is their purpose? One might further question why anyone would pay a premium to “own” an asset online if it's the kind of thing (an image, gif or clip, for instance) that one could just download for free^82^.

There is a mismatch between NFTs' ostensible capacity to confer uniqueness and authentication and what they actually can and do confer. This fundamental point about NFTs suggests that people who purchase them desiring to own something unique or rare may misunderstand the nature of NFTs as well as their rights in them^83^. To repurpose my earlier metaphor, evidence suggests that some buyers of NFTs may believe they are getting a piece of the luggage, a mistake that arises from conflating the luggage tag with the luggage contents^84^ even though NFT purchases rarely do convey more than the luggage tag. With one area of exception,^85^ neither the tag nor the luggage itself usually includes the original work as fully constituted, and certainly the NFT does not include any of the exclusive rights to it, unless—as Fairfield points out—the contract so specifies^86^. Even where a hash of the original work is “included” in a token (i.e., could be considered part of the luggage's contents), that hash merely contains a digital combination of numbers. With most kinds of NFTs, the work associated with the token is *not* uploaded and stored on the blockchain due to the high (technological and economic) costs of doing so^87^,^88^. Indeed, ownership of the token ordinarily conveys no other rights in the underlying asset referred to or stored in connection with the token.

The point of NFT is *not* ownership of the asset with which it is associated; the point of the NFT is to capitalize on the appetite for cool, unique luggage tags “worth” thousands or millions of dollars for certain buyers in connection with particular assets. Yet the popular perception misaligns with what owners actually buy if they purchase an NFT believing they are guaranteed the uniqueness and authenticity of an item they own and thus can control. As one skeptic put it, “It's like a ‘Certificate of Authenticity' that's in Comic Sans, and misspelt” (see text footnote 54).

Nonetheless, buyers continue to flock to this form of digital art “ownership”: the market for NFTs was valued at $41 billion in 2021, a figure nearly as high as the worldwide market for fine art. The market “stabilized” with an initially rough start in 2022 but by May 1, more than $37 billion had been spent in NFT marketplaces^89^. Predictions of a bubble bursting have thus not yet proven robust, and until the recent slide of cryptocurrency's valuation projected earnings had remained high^90^.

While there are ways to disseminate information and issue disclosures to clarify the precise terms of NFTs, the speed of transacting and the general hype around the market for NFTs may make it difficult to manage the associated risks of material misinformation. Perhaps this is a case in which buyers must simply beware, but if evidence suggests that many buyers are purchasing assets without an understanding of what they are and are not purchasing, perhaps these costs ultimately generate diverse losses that ought to give policymakers pause.

Third, NFTs bear *hidden maintenance costs*. Few purchasers appear to be considering what will happen to their token if the platform from which they purchase it ceases to exist. A number of NFT marketplaces are centralized platforms offering for sale a token stored on the blockchain whose asset is “stored off-chain”^91^ Because storing large digital files is costly, the asset to which the NFTs token refers is commonly not stored on the blockchain^92^. Yet this creates a potentially risky dependency: “if a NFT platform relies on a centralized server that stops operating the art, metadata, or media associated with that NFT may be lost forever”^93^. In many cases, maintenance or storage of the asset related to an NFT is not a guaranteed aspect of the transaction yet failing to maintain it would likely extinguish the legal interests as a practical matter^94^. One art history professor, interviewed back in 2017, stressed the risk of owning art that depends on the continued functioning of digital links^95^. Five years later, a fan of NFTs expressed concern that the risks of storage failures still have not been systematically addressed^96^. Companies have sprouted up to offer “pinning” services that guarantee to maintain an NFT's storage, causing owners of NFTs to assume ongoing costs in exchange for this peace of mind^97^. However, in both the rapidly evolving art market and the world of tech startups, the longterm viability of such entities presents its own risks, even if owners are willing to pay ongoing and unforeseen storage or “pinning” costs to hedge their NFT-storage bets.

The potential growth of NFTs thus carries multiple risks in the form of uncertainty, confusion and maintenance costs. These costs could conceivably be offset as the market for NFTs matures and new legal norms and practices take shape. Whatever one thinks of NFTs—aesthetically, sociologically, or legally—however, there is a cost that should not be overlooked until all NFTs adopt less resource-intensive technologies: *environmental costs*.

Journalists and scientists have widely documented the intense energy needs associated with distributed-ledger technologies, including those used for the majority of NFTs, such as Ethereum and Bitcoin^98^. As one commentator put it, “perhaps the only thing hotter than NFTs at the moment is, uh, the Earth.”^99^ The actual impact of cryptocurrencies is disputed but is often translated into the impact of various individuals, entities or nations. For instance, Ethereum reportedly consumes as much energy as Libya^100^; Bitcoin alone requires more energy than Finland^101^; Bitcoin mining around the world allegedly consumes more electricity annually than Argentina^102^; “a single Bitcoin transaction uses the same amount of power that the average American household consumes in a month;”^103^ and “minting artwork on the blockchain uses somewhere between weeks, months, years, (and in rare instances *decades*) of an average EU or US citizen's energy consumption.”^104^ Various sites permit interested users to calculate the energy impact of crypto art but such tools tend to underestimate the impact by counting only the energy required to track activity on the blockchain and omitting the costs of production, storage and hosting of the works connected to the NFTs (see text footnote 54).

Another way to assess the impact of certain cryptocurrencies is in terms of impact on climate and health. A team of scientists published a study in 2020 with their finding that “in 2018, each $1 of Bitcoin value created was responsible for $0.49 in health and climate damages in the US and $0.37 in China. Put differently, the human health and climate damages caused by Bitcoin represented almost half of the financial value of each US dollar of Bitcoin created (as represented by market prices.”^105^

This dire picture is unsurprising to those familiar with the technology's affordances. NFTs minted using major cryptocurrencies like Ethereum and Bitcoin rely on what is known as “proof of work” to guarantee their security and accuracy (see text footnote 100). Proof of work operates by requiring that users (known as “miners”) solve puzzles in order to be permitted to add verified transactions (or a “block”) to the blockchain. Solving these puzzles is time-intensive by design: “using up inordinate amounts of electricity — and probably paying a lot for it — makes it less profitable for someone to muck up the ledger.” Consequently, this “energy hungry” system of secure recordkeeping via proof of work *creates* scarcity at the expense of the actual energy required to run “energy-guzzling machines” doing the necessary work (see text footnote 100). Indeed, to the extent that computers grow more efficient at solving the puzzles, the challenges will necessarily become more difficult; the inefficiency is part of the verification value this system is thought to impart.

While it is possible to mint NFTs on platforms that do not use cryptocurrencies reliant on proof of work, it is less common (see text footnote 54). Other technological and market solutions exist, including creating an intermediary or parallel chain to increase efficiency with respect to transactions on the primary blockchain; these may take various forms as a “side chain” or a “second layer”^106^ such as Bitcoin's Lightning Network.”^107^ Another option is to conduct transactions on a wholly private blockchain specializing in NFTs, such as Flow (see text footnote 54, 100)^108^. However, such moves reduce the appeal driving the technolibertarian hype in the first place, namely open and decentralized transactions (see text footnote 100)^109^. An improvement over proof of work would be for a wholesale shift to what is known as “proof of stake,” which requires a form of digital escrow: instead of verifying users' bonafides by requiring that they consume a certain amount of resources via mining (as proof of work does), users instead must ante their own cryptocurrency tokens as a “stake” they could forfeit under certain conditions, somewhat like a lien on a traditional asset (see text footnote 100). Ethereum had long promised that it would shift from proof of work to proof of stake yet industry insiders recognize that the challenges and risks of doing so could threaten the entire system (see text footnote 100). The alternative, however, is unpalatable; as one person has put, “proof of work places a direct lien against the future”^110^. In the final stages of publication of this article, Ethereum did indeed switch to proof of stake^111^. This heralds a promising era in which the environmental costs of at least some NFTs could be lowered but the net effect of this change and its ripple effects cannot yet be measured.

At least some artists working in this space have long recognized the significant environmental costs of NFTs. Some have advocated for boycotts and called NFTs an “ecological nightmare pyramid scheme” (see text footnote 100) One group of artists has pledged to make only carbon-neutral NFTs^112^. Others are more optimistic about the prospects of cleaner NFT production in the future^113^. But not all NFTs are minted by creators who adopt an environmentally critical account of NFTs. Moreover, claims that environmentally tolerable NFTs represent the future have been met with skepticism^114^. Nonetheless, NFTs consume an irresponsibly large amount of energy to generate an asset whose primary purpose is—on most platforms—to provide luggage-tag style identification.

Returning to the framing of this chapter, the fanfare associated with NFTs presents a fable of scarcity. It opens with a *spectacle of need*, diversionary panic over the inability to locate authenticity or uniqueness in our digital moment, paired with concern over the inability of artists to reach audiences and monetize their art because of intermediaries who structure and may throttle transactions in digital art. “Piracy” also looms as an existential struggle even when there is scant empirical data in evidence that infringement—rather than changing behaviors and market factors—imperils artistic survival. The conflict or struggle involves a plethora of platforms and channels failing to meaningfully connect artists with willing buyers, thus exacerbating musicians' inability to make a living in an era in which online streaming rights favor platforms and large entities rather than artists. The *rallying cry* then is that minting NFTs will restore some sort of utopian abundance; perhaps the abundance of aura or specialness in art or perhaps the idea of an independent artist able to live off their art and fans able directly to support the work and artists they love. This blend of nostalgia and futuristic idealism drive the scarcity fable and compel its conclusion: *more property*.

Yet the scarcity fable also actively conceals the costs of this particular solution, including uncertainty, confusion, maintenance and environmental costs. In particular, the NFT scarcity fable masks the *actual scarcity* of environmental resources spent in profligate fashion in the minting of most NFTs. In the name of artificial scarcity portrayed as part of scarcity-mongering industry accounts, the triumphalist account of NFTs conceals and threatens to exacerbate actual scarcity.

## Artificial scarcity as rallying cry (solving for property)

In the IP context, scarcity fables may take diverse forms of scare mongering, or “scarcity mongering.” Scarcity mongering may operate as a call to propertize in domains of abundance in which the purported “scarcity” pertains to available rights rather than subject matter. For instance, it has been widely observed that trademarks for beer names have grown acutely scarce^115^. Yet framing this phenomenon as a problem may obscure possible benefits associated with such trademark scarcity^116^. It also focuses attention on the lack of capacity to create exclusive rights in beer names rather than the shrinking public domain with respect to simply naming beers without propertizing those names^117^. Scarcity mongering may also serve as a multiplier for damages, as in the case on which this section will focus. The core elements, again, are the assertedly spectacular need, along with diversion away from other forms of actual scarcity, mobilized in service of a plea for more or stronger property rights. *Leonard* provides an extended example of the potentially significant and detrimental impact of such propertarian rhetoric in the context of wildly inflated valuations of the two photos the defendant infringed.

*Leonard v. Stemtech* featured a decade-long dispute between Andrew Leonard, a professional photographer, and Stemtech, a multi-level marketing organization that sold nutritional supplements^118^. Stemtech and its members used two of Leonard's images without authorization, beyond the scope of Stemtech's licensed use and repeatedly^119^. At the conclusion of a 4-day trial in 2013, a jury found direct, contributory and vicarious infringement and awarded actual damages in the amount of $1.6 million (see text footnote 119). Various aspects of the ruling—and the verdict's later affirmance on appeal—reflect departures from well-settled precedent and dramatize how scarcity rhetoric can play a problematic role in an over-expansive assertion of property rights. The *Leonard* saga offers an example of a scarcity fable playing out in the context of copyright doctrine. It also provides an opportunity to focus on how a scarcity fable moves from the spectacle of need to the proposed propertarian resolution.

In 2008, Leonard sued Stemtech based on their unlicensed use of his photographs of human bone marrow stem cells^120^. At the time, Leonard was one of only a few photographers engaged in stem cell photography, a highly technical art form which uses electron microscopes to capture images of stem cells^121^. Leonard paid scientific research institutions for the use of their microscopes to deliver images in black and white^122^. Leonard then added color using his “artistic judgment.” Leonard marketed his photographs through multiple channels, including via a stock photography agency, Photo Researchers, Inc. However, he permitted only limited licenses because “in his view, unlimited usage licenses decrease the value of his work” (see text footnote 122). At the time of the photos' creation, stem cell images were rare and few photographers possessed the skill to capture them (see text footnote 122). Leonard's licensing fees ranged from hundreds of dollars per image up to $6,500 for one 4-year use of an image on a university website^123^.

Defendant Stemtech produces and sells nutritional supplements “through thousands of distributors who form the backbone of the company” (see text footnote 123). In 2006, Stemtech sought a license of one of Leonard's images. Leonard quoted a price of $950 for a 1-year license to use an image in two places in Stemtech's internal “HealthSpan” magazine and $300 for a 1-year license for use on Stemtech's website. Stemtech declined the website license but used the image twice in its magazine. Stemtech also used his images in multiple promotional materials without permission or license. In October 2007, Leonard discovered widespread unauthorized uses by Stemtech and/or its affiliates that continued through May 2008 despite Leonard's notice to Stemtech and his ongoing documentations of unauthorized usage. Leonard's request that Stemtech and several of its distributors pay him for their unauthorized use was refused, prompting Leonard to sue for copyright infringement (see text footnote 123). In the first phase of litigation, *Leonard I*, a magistrate judge ruled that Leonard was ineligible to seek statutory damages^124^. In *Leonard II*, the court granted defendants' motion for summary judgment with respect to disgorgement of “indirect profits,” ruling that the plaintiff had failed to meet his burden of alleging a causal nexus between defendants' infringement and profits^125^. Stemtech's infringement of two images was undeniable, so the effect of these rulings was to concentrate the subsequent trial in *Leonard II* on (1) whether Stemtech could be held secondarily liable for the acts of its distributors and (2) how expansively to calculate actual damages.

Trial began, during which a jury heard expert testimony to support Leonard's proposed calculation of damages. Under Section 504(a) of the Copyright Act, a copyright owner whose work has been infringed may recover *either* (1) “actual damages” suffered as a result of the infringement, plus “any additional profits of the infringer” not already counted under the owner's actual damages, *or* (2) statutory damages^126^ Punitive damages are not available under the Copyright Act^127^. While undefined in the statute, “actual damages” has been interpreted to mean “any harm … suffered by reason of the infringer's illegal act”^128^ or “the extent to which the market value of the copyrighted work at the time of the infringement has been injured or destroyed by the infringement.”^129^ Damages amounts may not be determined by “undue speculation” (see text footnote 129). Case law reflects that there are two primary modes of setting licensing fees for the purposes of determining an actual damages awards.

First, damages can be calculated based on the fair market value “the owner was entitled to charge for such use.”^130^ To the extent that value drops as a result of the infringement, or that defendants profit from their infringement, those deltas in value can be factored into damages award (except insofar as they would be duplicative of each other)^131^. Second, damages can alternatively be calculated based on the owner's past licensing history^132^. Both methods are accepted, but in some instances, the choice of methods produces wildly diverging amounts, which would prove to be the case here^133^.

Leonard's expert, Jeffrey Sedlik, began by gathering information to create a market benchmark. He explained to the jury that he had contacted general stock photo agencies as well as two that specialized in scientific images. To derive fair market value for Leonard's work, Sedlik began with an estimated licensing fee roughly between $1,200 and 2,600 per image for media uses such as Stemtech's^134^. He averaged this licensing fee and multiplied it by 92, the number of infringing uses that Leonard asserted had been identified by the time of trial, to arrive at a proposed initial number of $215,767.66 (see text footnote 134). Sedlik then recommended increasing that figure due to the “scarcity or rarity” of the images and their “exclusivity,” since scarcity is “a factor that is considered in licensing”^135^. Sedlik first increased the license fee by a “scarcity premium” of 3–5 times the benchmark (see text footnote 135). Next, he added an exclusivity multiplier, for a further premium of 3.75–8.75 times the benchmark. Sedlik reasoned that “ ‘overuse or broad use' of an image … diminishes the value of other uses,” and since Leonard purportedly preferred to exercise more limited licensing rights, this preference justified application of an exclusivity premium (see text footnote 135). Consequently, Sedlik projected that damages should fall between $1.4 and $3 million.

*Leonard* provides an example of a scarcity fable with respect to its damages award. Hence it is helpful to reverse-engineer how its damages award came to be. Under one view, the award could be attributed to both underlawyering and overlawyering. Stemtech did not offer its own expert and apparently failed to vigorously cross-examine Sedlik about his use of these premiums^136^. By contrast, Stemtech clung to its argument that the award should be limited to $1,804.00, based on Leonard's past licensing history (see text footnote 137). Read in retrospect and given that the trial would hinge on actual damages, Stemtech's theory of the amount of damages seems just plain lazy, especially compared with the detailed evidence offered by Sedlik. Sedlik had testified in 15 prior copyright trials and appears well-versed in how to handle both lawyers and jurors. (As an example, Sedlik described his expertise in scientific photography with a jury-pandering reference to his virtuous boyhood: “I'm a microscope buff. I have a collection of microscopes at my home, vintage from the 1800s and early 1900s, from the 1700s. I've been making microscope photographs *since I was a Boy Scout*, so for quite a long time”^137^). Another bad fact for Stemtech's theory of damages was that one of its own executives described Leonard's images as valuable and stated under oath that he suspected the photos had helped sell their product^138^. Indeed, to prove vicarious liability, Leonard had to prove that the infringement would benefit Stemtech. Thus, the jury repeatedly heard some version of the claim that Stemtech's employees acknowledged that “images of stem cells lend legitimacy to products that purportedly enhance stem cell production.”^139^ A Stemtech customer with a Ph.D. swore the images had played no role in her decision making, thereby offering highly credible countervailing testimony. Still, the notion of the images' value having been purposely expropriated for direct commercial advantage lingered throughout the trial^140^.

Given this foreseeable line of reasoning and its potential relevance for damages, Stemtech could and should have done more to anticipate and account for the likely financial impact of these many allegedly infringing uses. Instead, Stemtech's proposed award of $1,804.00 amounts to just 1.19% of Sedlik's starting position, before multipliers^141^. A number somewhere between these extremes might have struck the jury as more reasonable and avoided the ultimately excessive verdict the jury returned. However under-lawyered Stemtech's position was on this question, the arguments in favor of the vastly higher award proposed by Sedlik are tautological and self-serving; they might be considered over-lawyered. There are at least five ways in which the damages figure was problematic, considered in terms of doctrine, logic, policy, fairness and scope of copyright protection. Sedlik's testimony was especially misguided and damaging with respect to its use of exclusivity and scarcity multipliers.

First, doctrinally, Sedlik erred by conflating objective and subjective approaches to licensing fees. *Leonard III* recognizes that “[f]air market value is often described as ‘the reasonable licensing fee on which a willing buyer and a willing seller would have agreed for the use taken by the infringer.”^142^ The standard (“reasonable licensing fee”) is objective not subjective, which differentiates it from the subjective tailoring of the past licensing history approach^143^. Recall that the court had stated of Leonard that “*in his view*, unlimited usage licenses decrease the value of his work.”^144^ Tailoring the images' fair market value to the plaintiff's particular “view” or his preference for exclusivity converts the objective standard to a subjective one. The very notion is self-serving as the court almost seems to call out with the clause, “*in his view*.”

Adopting this subjective perspective permitted Sedlik to focus attention on Leonard's purported preference for exclusivity, and provided cover for the idea of an exclusivity premium. To the extent the court actually adopted a subjective approach tailored to *this photographer* rather than to the *reasonable photographer* under the objective approach, it shifted from the fair market value approach to the past licensing approach. Accordingly, it is unclear why the award was not based on subjective evidence, which would have included Leonard's (much lower-value) past licensing fees rather than objective evidence (hypothetical figures drawn from stock photo agencies' data). Sedlik knew and interviewed Leonard's competitors in the specific field of stem tech and admitted at one point that their fees were considerably lower than the base fees he proposed at trial, yet he chose to exclude those figures from his calculations and instead used higher fees based on stock photographs not specific to science or stem-cell photography (see text footnote 144). Effectively, Sedlik cherrypicked his figures and conflated the two methods for determining the plaintiff's actual damages, thus improperly broadening the scope of possible damages.

Second, logically, if treated as generally valid reasoning, Sedlik's exclusivity argument would provide all plaintiffs in copyright cases with a perverse incentive. For instance, to explain his exclusivity premium, Sedlik was asked whether infringement could affect a photo's value. He answered yes, and his answer reflects how his thinking double-counts the impact of infringement on estimations of damages:

A good example would be let's say I have photos on my website and somebody comes and takes that photo and puts it on the cover of a book, and the book is published. I didn't know it. I didn't license it. Now no other publisher will use my photo on their book, so they've just robbed me of my exclusive right to license that image for usage on a book cover. Similarly, if they put it on T-shirts or if they use it extensively, *the value of my work can be depleted by unlicensed use*. In addition, of course, *I don't get the fee* that I would have received for that usage. I don't have the opportunity to negotiate that usage^145^.

Sedlik points to the delta in value caused by “unlicensed use” and Leonard's loss of licensing fee as losses justifying an exclusivity premium. Quite plainly, these are the very injuries that awards of actual damages are intended to remedy, namely, losses in the fair market value of a work presumptively caused by infringement^146^. Hence they should not be considered *extra* or in some way serve as evidence that an exclusivity premium should be applied. In most cases, a suing plaintiff can plausibly state that they did not and would not have authorized the infringing use. Thus, a successful plaintiff would always automatically qualify for enhanced damages simply by stating that any unauthorized infringement decreases the value of their work. Permitting an “exclusivity multiplier” on top of recovery for infringement creates a windfall for the plaintiff, as other case law has acknowledged^147^.

Relatedly, application of an exclusivity multiplier is tautological. Sedlik never confronts, and the court never answers, why evidence of few licenses or particular levels of supposedly limited usage should be taken as evidence of an “exclusivity” preference rather than as evidence of insufficient willing buyers at the pricepoint quoted. Perhaps the market would not bear the prices at which Leonard wished to sell the photos; if so, this is not evidence that should be used to augment the fair market value. On the contrary, it would offer evidence that the true fair market value is much lower than represented after application of an exclusivity multiplier. The exclusivity premium here also underscores the subjective, rather than objective, nature of the award's tailoring by emphasizing what *Leonard* would have preferred to charge vs. what *the reasonable photographer* would have been capable of charging.

Third, Sedlik's exclusivity multiplier subverts copyright law's remedies regime by effectively providing an end-run around the lack of punitive damages in copyright law^148^. The only means of recovering supracompensatory damages under copyright law is by seeking statutory damages and also proving willfulness on the part of the infringer^149^. In cases such as this one, where the plaintiff has not timely registered his copyright, statutory damages are unavailable^150^. The mere unavailability of statutory damages does nothing to change the lack of an alternative punitive damages system^151^. Outside of the statutory damages framework, courts have consistently held that the use of multipliers is not permitted, as *Leonard III* acknowledges but sidesteps^152^. Indeed, Sedlik himself was aware of this prohibition on multipliers, having taken that very position in prior litigation (as *Leonard III* notes)^153^. Unless such multipliers are included in the licensing terms to which parties agree, the use of multipliers to enhance damages does not comport with copyright law, as Sedlik's testimony in that earlier case correctly acknowledged^154^.

Enhancing damages outside of the context of statutory damages in a case in which the plaintiff fails to qualify for statutory damages further undermines the Copyright Act's balance of incentives and rewards. Leonard was an experienced professional photographer who routinely enforced rights in his works. He possessed sufficient skill and experience to have had his work selected for the cover of Time Magazine. By choosing not to register these two ostensibly scarce and valuable images, he failed to comply with a basic requirement for anyone who might wish to seek supracompensatory damages. Awarding Leonard supracompensatory damages as though he had registered and could qualify for enhanced statutory damages vitiates the registration requirement for this heightened remedy.

Fourth, in positing 92 infringements, Sedlik's estimate may have overcounted the instances of infringement. According to Stemtech's post-trial arguments, Sedlik counted identical uses of the images:

[E]ach time the *identical* e-book, or PowerPoint presentation or website is identified, Sedlik counts it as a separate and distinct infringement even though it is well-established that “[a] single infringer of a single work is liable for a single amount..., no matter how many acts of infringement are involved in the action and regardless of whether the acts were separate, isolated, or occurred in a related series.” … Sedlik's computation of Leonard's “actual” damages in this way is analogous to him finding a separate and distinct infringement in a situation where an image is infringed upon by placing it in a magazine or a video and then counting *every single copy of that magazine or video as a separate infringement*. Such a computation is contrary to law^155^.

This alleged overcounting of purported instances of infringement reflects that Leonard's strategy at trial was to hint that there were likely many more infringements than had been discovered, presumably because doing so would convey that the impact of infringement on Leonard was greater than the two infringed images at issue might suggest.

Shortly before trial, the court denied defendants' Daubert motion^156^ and motions in limine to exclude Sedlik's testimony on various grounds^157^. The court stated its initial sense that Sedlik's testimony seemed to lack a factual foundation but nonetheless thought trial would be the more appropriate time to assess the testimony. However, it did strike one statement of Sedlik's statement as “unduly speculative,” namely his belief that the infringements known to Leonard were likely only “the proverbial ‘tip of the iceberg.’”^158^ In his opening statement, plaintiff's counsel nonetheless told the jury that “Mr. Leonard had only discovered the tip of the iceberg,” apparently intent on conveying this message to the jury even without the expertise Sedlik might have lent such a statement^159^. In his testimony, Sedlik also found a creative way to raise the specter of innumerable undiscovered infringements without using the stricken statement:

You have to understand, Counselor, there is also the factor in this case that these are only the usages that Mr. Leonard discovered. And looking for usages on the internet is kind of, if you can imagine, an endless field of haystacks.” (see text footnote 145)

Sedlik's reference prompted an objection from Stemtech's counsel on the basis of the court's earlier ruling, which the court sustained^160^. Leonard's counsel's rhetoric makes it clear that the trial strategy was to amplify the number and value of the instances of infringement of these two works, regardless of the logical and doctrinal contortions involved in doing so.

Fifth—the court never engages with a troubling consequence of this over-expansive enforcement. Photography of stem cells, by its nature, is highly factual and low in originality. Copyright does not protect scientific data or basic facts, no matter how beautifully they may be presented^161^. Instead, what is protectable are the very aspects of the image arguably less likely to fetch top dollar. To the extent that Leonard certainly added some original touches, such as by adding color to enhance the images, these images' value came not from such after-effects but from the scientific information they communicated and the vivid, accurate way they communicated it. Leonard's skill allowed him to capture these elusive and valuable images, as the magistrate judge acknowledged in an early ruling: “[Leonard's] subject matter is often difficult to procure and prepare and, consequently, his photographs are highly desirable, particularly to the medical and pharmaceutical industries.”^162^ Indeed, at trial it emerged very clearly that the photographs were valuable precisely because they were scarce^163^. In turn, their scarcity was attributable to their value as artifacts that conveyed and represented hard-to-access scientific information at a time of growing interest in stem-cell research. Their value came, in other words, from their scientific nature, not their originality. Precisely because their value is informational rather than expressive, these images ought to receive only thin copyright. If the scope of protection in Leonard's works is interpreted broadly, the net effect of this is to allow copyright protection to drive up the cost in informational works despite their ostensible exclusion from copyright protection.

In spite of these five categories of contradictory or irregular reasoning, Sedlik's testimony seems largely to have been accepted by the jury and left undisturbed by the trial court^164^. After the jury returned its verdict for Leonard, defendants sought post-trial relief and the parties sparred over various issues (see text footnote 164). Stemtech moved for prejudgment interest which the court denied^165^. Stemtech subsequently moved for a new trial or remittitur on multiple grounds relating to the damages award and Sedlik's testimony, arguing in relevant part that the damages award was unconstitutional and grossly excessive^166^. Stemtech raised specific concerns over the jury's use of “scarcity and exclusivity multipliers,” given that other courts have consistently rejected the use of such multipliers^167^.

The trial court agreed that the damages award was excessive and cited to the reasoning from its own earlier denial of prejudgment interest: “The jury's $1.6 million verdict *more than fully compensates* Plaintiff for the misappropriated value of his property. As Plaintiffs expert witness, Professor Jeff Sedlick, testified at trial, $1.6 *far exceeds the aggregate value Plaintiff ‘received for all of [his] 92 previous licenses* Photo Researchers obtained over a 15–year time period for the use of the Leonard's Image 3 or 4…'.”^168^ To drive that point home: the jury's award was *100 times higher* than the $16,000 Leonard had earned during that entire 15-year window for licensing the two images in question; his average fee for commercial use was < $400 per image (see text footnote 168). The court actually noted that “the license amount implied by the jury's verdict is an average of approximately $17,000 more per infringing use than Leonard's average commercial license fee actually obtained by Photo Researchers” (see text footnote 168). Finally, in their negotiations, Leonard had priced Stemtech's licenses in the hundreds, not thousands or millions, underscoring the vast disparity between what appeared to be the images' actual market value and the jury's damages award (see text footnote 168). The trial court thus concluded that the award was excessive.

However, the court held that the award, though excessive, was not “unreasonable” in light of the evidence the jury heard (see text footnote 168). In denying Stemtech's motion for a new trial, the court took note of various testimony before the jury, including the opinion offered by Leonard's licensing agent, Mr. Gerard. Gerard had attested to the rarity, beauty, popularity and thus high value of Leonard's images, noting that the demand “was a lot higher for Leonard's material, just because of the subject matter.”^169^ Despite the court's finding that the award was excessive nature, it ruled that the award did not provide grounds for a new trial.

Stemtech appealed and the United States Court of Appeals for the Third Circuit upheld much of the lower court's decision but held that the jury award was not excessive^170^. The court held that “[b]ecause the jury was instructed about both methods for determining actual damages, and had an evidentiary basis for applying the fair market value through Sedlik's expert testimony, there was no error.”^171^ Stemtech continued to argue in vain that the award's inclusion of multipliers rendered it excessive and improperly punitive (see text footnote 171).

The weight of legal authority seemed unquestionably on Stemtech's side of the issue, casting doubt on the soundness of *Leonard III's* holding. Other courts have consistently ruled that adding multipliers in the calculation of fair market value impermissibly expands damages awards. For instance, in a 2004 case, *Stehrenberger v. R.J. Reynolds Tobacco Holdings, Inc*., a graphic artist sued over the unauthorized use of her artwork, “Blue Girl” to market cigarettes^172^. Defendants had copied Stehrenberger's work, airbrushed out her signature and copyright symbol, changed the color of the image to sepia, reversed the image, and added the company's “Camel” branding to headphones worn by the girl depicted in the original work^173^. Stehrenberger's counsel proffered expert testimony of an industry practice apparently known as “retroactive licensing,” or licensing after the discovery of infringement^174^.

The expert, Henri Dauman, testified that industry guidelines applied a multiplier in such cases, to signal disapproval of infringement and to avoid results that would effectively seem to sanction infringement. According to Dauman, an appropriate licensing fee for “Blue Girl” negotiated ex ante might have been $60,000 for the use of the image in national tobacco advertising; the same use negotiated ex post would command $600,000 (see text footnote 174). Dauman testified that it was “a Media Industry standard to seek permission” prior to use but that in cases of “mistakes … resulting in unauthorized use,” the common practice was to attempt to “resolve the violation amicably and early on” via “retroactive license for a fee, which is recommended as three times (3x) the normal fees, … when the infringer recognizes the ‘mistake' and moves quickly to correct it” (see text footnote 174). In cases in which the infringer does not act expeditiously, the multiplier purportedly jumped from three to up to ten, Dauman reported. He explained the reasoning behind this practice:

A potential licensee who makes an effort to negotiate a fee prior to the use is in a different position from one who appropriates the image outright and tries to get away without paying. The Copyright Law does not condone a practice of ‘infringe now, pay later.'…[Because otherwise] “[t]he industry would have no incentive to bargain for a fee prior to using an image, and therefore the enforcement of copyrights would have no teeth…. The plaintiff cannot be a policeman. If a friend had not recognized the unique Blue Girl Image, [defendants] might have gotten away with it” (see text footnote 174)

Dauman's use of multipliers unmistakably conveys an intent to deter and punish (“The plaintiff cannot be a policeman”), rather than merely compensate. Dauman further opined that a 10-fold multiplier in this case was “conservative” in light of practices in the graphic arts community and would not provide the plaintiff with a windfall (see text footnote 174)^175^. His opinion flowed in part from his view that the usage was “clearly without consent and … clearly willful,” yet this introduces an element not appropriate to the analysis of damages outside the context of statutory damages, where willfulness may be considered^176^.

The court correctly rejected Dauman's view, observing that “[w]hatever its utility as a marketplace technique for resolving problems among the ‘graphic arts community,' this claimed practice is not the method by which damages are calculated under the copyright law” (see text footnote 176) Estimating market value under Dauman's view would clearly incorporate “concepts of punishment for infringement, deterrence of similar behavior in the future, and recompense for the costs and effort of litigation” (see text footnote 176). Yet such behavioral levers “form no part of ‘actual damages' under the statute” (see text footnote 176). Again, while the Copyright Act permits plaintiffs to seek enhanced damages, it does so only under its statutory damages regime; the Act contains “no provision for ‘multipliers' in the calculation of actual damages.”^177^ Moreover, such a multiplication would distort the assessment of value since “infringement does not make a copyright more valuable” (see text footnote 177). As the court emphasized:

The “value of what was illegally taken” is not determined by multiplying it. Plaintiff's expert calculated that value at $60,000 and (if that figure is proved) that amount, and not a multiplication of it, represents plaintiff's “actual damages.”^178^

*Stehrenberger* demonstrates the illogic and impropriety of using the fact of infringement to retrospectively ratchet up the fair market value of the work for the purposes of determining actual damages. Nonetheless, subsequent case law demonstrates that despite that illogic, attempts to introduce such multipliers persist.

In *Faulkner v. National Geographic Society*, decided 4 years after *Stehrenberger*, Dauman—the same expert as in *Stehrenberger*—again testified about “industry guidelines” and the 3–10x multipliers based on the parties' conduct^179^. Again the court declined to adopt his testimony. In a sharply worded opinion, the court dispensed with the theory that multipliers could be used to increase the fair market value for the purposes of determining actual damages. In a footnote, the court expressed doubt about the interplay between the alleged norms of the graphic arts community and the formal processes of litigation: “Nowhere does [Dauman] explain how ‘the industry' applies a multiplier of up to 10 times where the photographer goes to court. All of such cases presumably are resolved by adjudication or settlement between the photographer and the alleged infringer.”^180^ In a more direct assault on Dauman's use of multipliers, the court noted once again the clearly punitive character of such multipliers: “The basis for Mr. Dauman's six times multiplier is his personal view that ‘National Geographic basically stole the crown jewels from these photographers without paying anything. … The use of such a multiplier is simply a vehicle for punishing the publisher.”^181^

Of particular relevance in understanding *Leonard* is the court's critique of Dauman's use of a multiplier for unauthorized use:

The application of a *multiplier … for the use of an image without authorization*—in other words, *for infringement*—is purely punitive and entirely improper. It certainly is not anything that would have been agreed between a willing licensor and a willing licensee. …Indeed, *this entire portion of his opinion is constructed on a base of sand*. It starts with an unsubstantiated assumption concerning an initial press run limitation and proceeds by nothing more than guesses about multiple renewals or modifications that, in reality, are excuses to increase his $1,350 base fee at a compound rate, each baseless step based on the preceding guess^182^.

*Faulkner* illustrates that a multiplier for unauthorized use is duplicative of the standard remedy for infringement; the need to correct for harm associated with an unauthorized use is literally the core purpose of actual damages and need not be separately factored in a second time. Building an additional premium in for unauthorized use—like Sedlik's “exclusivity premium” in *Leonard* —improperly inflates a damages award as a form of punishment rather than as a measure of market value. Dauman's efforts also seem like a backdoor attempt to introduce prejudicial willfulness evidence even though defendants' intent is irrelevant in calculating damages and copyright is a strict liability tort^183^.

Consequently, considerable authority holds that “actual damages” are limited to the fair market value of a license as that would have been determined before infringement^184^ and numerous courts have held that actual damages *must not include* multipliers that increase the award simply due to the fact of infringement^185^. Indeed, *Faulkner* stated that “arguments substantially the same as Mr. Dauman's have been rejected in every case to consider the question.”^186^ Dauman's testimony in *Stehrenberger* and *Faulkner* referred to notions of deterrence and punishment which are facially missing from the Act and thus may have been straightforward to identify as error. Similarly, in cases in which the parties speak openly about the punitive aspects of proposed damages awards, error is easier to identify since punitive damages are unavailable as such under the Copyright Act^187^. The Nimmer treatise identifies a lone case to the contrary, in which a court denied defendants' request for a jury instruction that would have stated that punitive damages were categorically unavailable under the Copyright Act. Noting it as a “rogue decision,” Nimmer recommends against following it^188^.

The reigning view, at least before *Leonard III*, was thus that multipliers to enhance actual damages awards were unavailing and improper; statutory damages provide the only mechanism for enhancing damages under copyright law and even these should not be characterized as “punitive damages” *per se*^189^. This treatment of multipliers is consistent with their treatment in other areas of law, where punitive damages are often greeted with skepticism (or even deemed unconstitutional). Copyright litigation confronted the issue of multipliers in the context of peer-to-peer sharing and while enhanced damages were ultimately held constitutional, the legal fight assessing this constitutionality was protracted^190^. The trial court in the *Leonard* litigation at least acknowledged the excessive nature of the award and foregrounded a more plausible justification—jury deference—in refusing to disturb the jury verdict^191^. However, it is puzzling how the appellate court could seem to sanction punitive multipliers.

*Leonard III* acknowledged that “the Act does not authorize recovery of punitive damages” but sought to explain away the contrary case law by ruling that the jury's $1.6 million award did not include punitive damages^192^. Instead, the court reasoned that Sedlik's multipliers were merely being used to determine fair market value, rather than being multipliers applied, ex post, to the fair market value determined ex ante^193^. In light of the compelling reasoning of prior courts on this question and the numerous deficiencies in Sedlik's reasoning, *Leonard III's* explanation amounts to an unsatisfying dodge. Again, as discussed above, the use of multipliers cannot be justified doctrinally (it improperly conflates subjective and objective approaches to actual damages) or logically (as its incorporation of an “exclusivity” premium would tautologically expand damages for any unauthorized use). It also undermines copyright policy, which explicitly omits punitive damages from copyright law and requires registration for those seeking enhanced damages under the statutory damages framework. Lastly, it distorts the scope of copyright protection by awarding an excessive amount of damages to a work possessed of very high skill but little originality, the sine qua non of copyright law^194^.

Including multipliers in the calculation of fair market value is an exercise in speculative accounting that imports illogical, self-serving and expansionist reasoning. Permitting use of multipliers imports a punitive element that, as noted above, copyright law otherwise expressly omits. Substantive rules and existing case law would seem to foreclose the outcome in the *Leonard* litigation. Yet at the heart of this dispute over a pair of photographs of stem cells lies a scarcity fable powered by Sedlik's testimony. It launches with a spectacle of need and seeks to restore abundance in the form of a supracompensatory damages award that the trial court even conceded was excessive. Throughout, notions of artificial scarcity prop up the rallying cry in a way that distorts legal reasoning and diverts attention from the true scarcity applicable to representations of data and scientific facts. Sedlik's rhetoric and storytelling demonstrate how a scarcity fable may resonate with jurors. A close reading of his testimony provides some insight into trial dynamics and illustrates the risks posed by scarcity fables.

Sedlik begins by establishing Leonard as a serious photographer and declaring that photographers face dire threats to their traditional business model.

Many people think that photographers earn their living by taking pictures or by selling pictures. They really don't. They earn a living by licensing the pictures, by licensing the copyright in the picture. They need to be able do that over and over again in order to be able to have the revenue to support their families and themselves^195^.

Sedlik's testimony frames the spectacle of need by emphasizing the importance of licensing to making a living. Sedlik conveniently sidesteps the documented numbers Leonard earned for these particular photos (< $16,000 over 15years)^196^. Instead, Sedlik focuses on the threat of infringement to the photographer and his family.

Next, Sedlik references Leonard's success in having one of the two photographs at issue in this litigation on the cover of a prominent publication. Leonard's counsel asks him to elaborate.

Nobody is going to make a living having their photograph used on the cover of Time Magazine. It's editorial use. It's very, very difficult to earn your living from only editorial use^197^.

Sedlike underscores the “very, very difficult” circumstances for even celebrated photographers, amplifying the spectacle of need.

Strictly speaking, that the licensing fee offered for a Time cover is unusually low is irrelevant to the legal discussion for two reasons. First, as noted above, the fair market value of the image uses an objective, not subjective approach, so the photographer's past licensing fees are not supposed to provide the benchmark for the award here. Second, to the extent that the Time licensing fee had any bearing on subsequent licenses for that image or Leonard's other work, it seems likely to *increase not decrease* subsequent revenues for Leonard. Such a placement is considered an unusual honor and an effective way to generate publicity for one's work. Photographers are likely to accept correspondingly lower fees for such high-profile works. Sedlik's point is not meant to bolster the legal arguments here, however, but rather aimed at bolstering the spectacle of need: skilled photographers like this one struggle to make money for their work.

With this spectacle in place, Sedlik lays the foundation for his rallying cry, a resolution aimed at abundance in the form of a damages award adjusted upward multiple times. He begins with scarcity or rarity (which he appears to use interchangeably). Sedlik explains that his award amount must take scarcity into account because it is not enumerated in the licensing menus used by stock agencies, which list factors that affect pricing ^198^. Sedlik fails to explain why its absence from this detailed enumeration is not proof that the image's rarity has already been factored into the pricing. Instead, he offers an elaborate story designed to explain why scarcity drives value:

But let me put it this way. If you're walking through the forest with a cell phone camera and a creature walks by. You take a picture, and you look and it's Bigfoot. You've got a very sharp picture of Bigfoot. You're going to be able to license that image for a considerable amount of money because it's extremely rare. There are no other sharp images of the alleged Sasquatch or Bigfoot (see text footnote 198).

Sedlik's remarks suggest that some photographs are rare on account of lucky timing, which is consistent with how some courts have analyzed copyright in photographs.

For example, in *Mannion v. Coors Brewing*, the court taxonomized the kinds of originality that give rise to copyright protection in photographs, including originality in rendition, timing and subject creation^199^. As one example of originality in timing, *Mannion* pointed to Thomas Mangelsen's famous photograph, *Catch of the Day*, which captures what appears to be a salmon jumping into the mouth of a patiently waiting brown bear^200^. Yet *Mannion* makes clear that protection derived from a photograph's original timing does not necessarily confer rights in the subject matter, even if cleverly captured. “[I]f another photographer were sufficiently skilled and fortunate to capture a salmon at the precise moment that it appeared to enter a hungry bear's mouth—…that photographer, even if inspired by Mangelsen, would not necessarily have infringed his work because Mangelsen's copyright does not extend to the natural world he captured.” (see text footnote 200)

Under *Mannion*, Leonard's rights to stem cell photography would be limited to the original elements he added and would not confer a monopoly in stem cell images. The issue was not central to litigation because copying the images was conceded and thus there was no need for an assessment of similarity, where the scope of protection in the work would have been centrally at issue. However, Sedlik's scarcity rhetoric hints at providing Sedlik with a market premium based on something not intrinsic to copyright law's threshold requirement of originality; the images' alleged scarcity had to do with the advancement of technologies associated with microscopes and photography. As functional advancements of the arts and technologies, they lie outside the purview of copyright, whose domain includes expressive contributions and excludes useful ones. It is thus telling how Sedlik conjures scarcity.

To dramatize the stem cells' scarcity, Sedlik unironically offers as the subject of this hypothetically rare photograph a mythical creature, literally impossible of being photographed because, like the Loch Ness monster, mermaids, or unicorns, it does not exist^201^. When he continues, Sedlik retreats from the impossibility of his own metaphor slightly by adding that photographs may be rare when they feature “certain public figures caught in certain situations, or celebrities who have passed away” since such photographs may be impossible to recreate. However, to the extent that these potentially-real examples point to photographs whose value is bound up with their newsworthiness, they may undercut Sedlik's expansionist reasoning. Case law suggests the opposite, in fact; to the extent that photographs (and other visual works such as films) are newsworthy, even when rare, they may be *more* available under fair use and thus potentially *less* protected ab initio^202^.

Curiously, Sedlik uses the Sasquatch as a recurring motif. Leonard's counsel asks whether license scarcity or rarity could affect the licensing fee a photographer would charge. Sedlik answers that “the scarcity or rarity of particular stem cell images” is “a factor… considered in licensing” ^203^ and elaborates as follows:

In the lower range, you have three to ten times the price of just an average image, let's say. In the upper range, you have *that Sasquatch effect*, where you have something that's just impossible or unlikely to create [sic] otherwise, and you can have 100 times or 1,000 times or just extraordinary numbers^204^.

The Sasquatch effect, as he coins it, seems to involve inflating the estimated value of a work on the basis of scarcity so acute it can only be captured via supernatural metaphor.

For example, that Sasquatch example that I made earlier. Stem cell is not Sasquatch; however, every photographer, everybody in the industry that saw that 2006 cover of Time, that was kind of a turning point where people realized that microscopy can be an art form. Previous to that, microscopy was viewed as something technical that technicians did to capture small things and make them appear larger, and after that, there was a lot of interest by many people, including myself, in making the art form. … I would say that at that earlier time, in 2006 and before, there were fewer images available.

Sedlik distinguishes Leonard's work but nonetheless continues to use the Sasquatch image to underscore the photographs' scarcity. Observe, too, how Sedlik converts the Sasquatch from a hyperbolic rhetorical flourish to an “example,” (“that Sasquatch example”) suggesting the slippery terms of his own argument. Even as he seems to acknowledge that “Stem cell is not”, like the Sasquatch, supernatural, his own deliberate and recurring juxtaposition signals that the rarity here is sufficiently similar to warrant the comparison. Indeed, by casting the Sasquatch as an impossibly rare figure whose “impossible or unlikely” photographic capture could justify premiums from three to 1,000 times an ordinary license fee, he seems to be asking the jury to believe in a kind of magic.

Accordingly, Sedlik offers the jury a means of providing Leonard with abundance according to the logic of a scarcity fable. Despite Leonard's lack of registration and the consequent unavailability of enhanced statutory damages, the jury has a role to play in correcting this injustice. In other words, it can help punish Stemtech and correct for the dire scarcity from which Leonard will otherwise suffer. To reiterate the obvious, however, such a photograph literally cannot exist (unless faked): the Sasquatch effect Sedlik is attempting to sell is a form of funny math or fake news belonging, like its namesake, to an epistemology of the unreal.

Sedlik's testimony may have been blessed on appeal, but it nonetheless can be seen as operating as part of the plaintiff's scarcity fable, driven in this case by the “scarcity” and “exclusivity” associated with Leonard's scientific images and a compelling story about the inability of contemporary photographers to make a living in a rough field of infringing and unfair uses. Only by understanding how fair market value here internalizes particular constructs of artificial scarcity can the ruling be fully explained in light of existing doctrines and precedent.

Besides departing from well-settled precedent against the use of inflationary or punitive multipliers, *Leonard III* could be read as creating a circuit split^205^. Notwithstanding *Leonard III's* contrarian reasoning, the Supreme Court of the United States refused Defendants' petition for a writ of certioriari and the case remains good law^206^. *Leonard III* has been cited 124 times in the 6 years since its issuance^207^. Some courts have distinguished its use of multipliers in ways that suggest *Leonard's* influence could remain limited to cases involving highly technical scientific images^208^. The notion of a “scarcity multiplier” has nonetheless appeared to have rapidly gained in popularity, which offers some correlative evidence of *Leonard's* influence. At least 37 federal courts have used the phrase in copyright rulings, and all but one outlier were decided after *Leonard III*, in 2018 or later^209^. Some courts that adopt a scarcity multiplier cite *Leonard* as justification, incorporating scarcity in determinations of fair market value without acknowledging the earlier case's highly specific scientific context and without looking in any detail at the problematic exclusivity premium^210^. Many cite to *Leonard* in the context of statutory damages, where the question of willfulness is actually relevant thus glossing over the infirmity of Leonard's own use of multipliers in the context of actual damages where enhanced damages are *not* permitted.

Perplexingly, given the reality revealed by the trial record, *Leonard III* is described by commentators as a case that affirms that (1) punitive multipliers are not allowed in copyright's actual damages regime and (2) multipliers may be permitted only in the determination of fair market value^211^. However, *Leonard III's* reasoning is flawed and tautological, as described at length above. It props up a questionable holding that effectively blesses punitive multipliers justified on grounds of scarcity and exclusivity, both as constructs that are not natural phenomena but inflated guesses, about as connected to reality as the mythical sasquatch Sedlik evokes. Furthermore, the very emphasis on these forms of artificial scarcity expands the owner's property rights and obscures the risks and costs of actual scarcity. There is no discussion of the impact on the public domain or other scientists and artists of protecting the work in question, let alone overprotecting it to the excessive, augmented level approved by both the trial and reviewing courts. *Leonard v. Stemtech* thus illustrates how a scarcity fable draws focus to restoring abundance in the form of greater propertization while minimizing or even suppressing robust discussion of the costs, in the form of actual scarcity, that greater propertization may impose.

## Conclusion

A scarcity fable can be used to create or strengthen property rights by painting a vivid picture of need and following it with a persuasive pitch to meet that need through a property-based solution: more enclosure, less need. In IP law, the scarcity fable may conclude with a call to propertize (with little corresponding attention to the risk of shrinking the commons or impeding competition and follow-on creativity). In some instances, as in this *Leonard*, propertarian rhetoric impels claims for multipliers to be applied to damages.

Yet fables of scarcity may displace or conceal the externalities associated with these purportedly happy endings. That is, they may gin up support for a solution to *artificial* scarcity and, in so doing, shift attention away from *actual* scarcity. Scarcity multipliers incorrectly applied in copyright's actual damages regime provide one case in point, as Leonard's pair of scientific photographs illustrated; the hype around NFTs offers another. Traditionally, the very way that most NFTs generate value is through artificial scarcity achieved primarily by extravagant consumption of resources^212^. In the name of “curing” the scarcity of the authentic or verifiably unique in the digital era, NFTs contribute to deepening our collective environmental crisis. In other words, to “solve” for artificial scarcity, NFTs worsen real scarcity. Part of the success of NFTs may lie in the rhetoric associated with selling them as a solution rather than a costly problem that merely produces the need for new solutions.

Scarcity fables reflect that scarce resources—dramatically depicted in the spectacle of need— “are necessary conditions, even if not sufficient ones” in “produc[ing] property regimes”^213^ Whether this scarcity is mapped onto a human body, a political domain at war, or a collapsing creative market, when the need is spectacular enough, it sounds a distracting note of alarm and impels the search for a solution. The rallying cry, through the rhetoric of restoring abundance, promises resolution and seems to offer an idealized answer to the questions posed by dramatic scarcity. Yet scarcity mongering is an exercise in question-begging and more exclusivity is thus almost always the “right” answer.

Attending to the constructedness of the scarcity fable in legal storytelling provides clues for interrupting such propertarian narratives and reframing their underlying questions at the outset. Artificial scarcity is a powerful motivator when the right kind of story about it is told, a scarcity fable whose originating conflicts and ultimate resolution entrench certain perspectives on consumption and ownership. To those with a propertarian mindset, everything may look like a potential parcel, an ownership interest to be defined, deeded and defended. The persuasive storytelling made memorable in fables of scarcity may form part of a campaign to propertize—to create, expand or strengthen property rights. Scarcity mongering operates within a logic of ownership that reifies property rights and obscures or devalues disappearing abundance elsewhere, such as in the public domain and the environment, where verifiable scarcity exists and may present truly existential threats.

The nature of this chapter is necessarily conceptual and speculative, designed to raise questions about different narratives of scarcity rather than attempting conclusively to answer them. Through juxtaposition of a handful of literary accounts and one legal case study, fables of scarcity begin to emerge as a possible genre whose very appearance in certain contexts ought to give scholars and policymakers pause. In copyright litigation, in which expansionist property narratives may be especially harmful to the public domain and subsequent creators, scarcity fables may be made to provide apparent support for potentially dangerous changes. Identifying scarcity fables as such when they appear in copyright cases could trigger review of the asserted scarcity and a more searching inquiry into whether the proposed solution could worsen actual scarcity.

## Data availability statement

The original contributions presented in the study are included in the article/supplementary material, further inquiries can be directed to the corresponding author.

## Author contributions

The author confirms being the sole contributor of this work and has approved it for publication.

## Footnotes

^1^Describing modes by which advertisers target their messages to land with optimal impact based on different personality traits, including in ways that sometimes deliver benefits to consumers (Calo, 2014, p. 1016–1018, 1030–1031).

^2^See e.g., Benjamin (1955) and Hansen (2008).

^3^Lemley (2015).

^4^Lemley (2015, p. 462).

^5^Rose (1998).

^6^“Every work of literature has both a situation and a story. The situation is the context or circumstance, sometimes the plot; the story is the emotional experience that preoccupies the writer: the insight, the wisdom, the thing one has come to say” (Gornick, 2001).

^7^Lemley (2015, p. 462).

^8^I adapt the name from the title of one of the germinal works of cultural history on advertising, a domain in which the rhetoric of scarcity has often been mobilized to stimulate desire and fear as a way of selling something (goods, services, a way of life) (Lears, 1994).

^9^Describing “the art of hunger” as a modernist trope later deployed by Coetzee “in dialogue with earlier modernists manifestations” including Kafka's A Hunger Artist and Melville's “proto-modernist” account of death by starvation; see *infra* for discussion of Melville's short story (Moody, 2018).

^10^Leonard v. Stemtech International, Inc, 834 F.3d 376 (2016), (“Leonard III”).

^11^Textual citations to A Hunger Artist indicated in the body of the text by “HA” (Kafka, 1924).

^12^West (1985, 1986), Posner (1986, 1988).

^13^West (1985, p. 393).

^14^Posner (1986, p. 1434).

^15^West (1986, p. 1452–1453).

^16^Taking Exception to the Exception (Summer - Fall) (Buelens and Hoens, 2007).

^17^Meyer (2014).

^18^Textual citations indicated in the body of the text by “LT” (Coetzee, 1983).

^19^Asserting that Coetzee's “source” for the corrugated-iron hole in which Michael K hides for a long period of time is Kafka's story, “The Burrow,” which Coetzee has analyzed in other writing). It is also supposed that Michael K's name gestures to Josef K, the doomed protagonist in Kafka's *The Trial* (Meljac, 2008, p. 70).

^20^Locke (2008).

^21^Moody (2018, p. 175).

^22^Compare Kafka: “An overseer's eye fell on the cage one day and he asked the attendants why this perfectly good cage should be left standing there unused with dirty straw inside it; nobody knew, until one man, helped out by the notice board, remembered about the hunger artist” (HA, 254) with Coetzee: “Well, we forgot about [K]” (LT, 154).

^23^Moody (2018, p. 3, 1, 158).

^24^Juxtaposing the two works and noting that “the motif of excessive hunger is used to mark a shift in an unspecified historical moment;” “not eating in the face of food availability marks food refusal as subversion and situates starvation as both an affront to and a withdrawal from dominant ideological ‘audience” (Wright, 2001).

^25^Linford (2020).

^26^K's stubbornness causes the Medical Officer to remark to his colleague, “It's not a question of dying… He just doesn't like the food here… Maybe he only eats the bread of freedom.” (LT, 146).

^27^Early drafts of the novel emphasized famine and material scarcity but by his fourth draft, Coetzee had shifted away from “collective, poverty-induced malnourishment” and into a more symbolic register that makes the novel's lineage within the “art of hunger tradition” (Moody, 2018, p. 175).

^28^Moody (2018, p. 181).

^29^Melville (1998), Moody (2018, p. 175).

^30^Relating how “the tale unfolds like a kind of experiment in which an inaccessible mind is dropped into a conventional nineteenth-century storyworld” (Abbott, 2008).

^31^See e.g., Brown (1989) and Desmarais (2001).

^32^“The narrator himself is driven to think that ‘the scrivener was the victim of innate and incurable disorder' (111–112). But, strangely, it doesn't work for him, and he must repeatedly return to the stubborn fact that “Bartleby was one of those beings of whom nothing is ascertainable” (Abbott, 2008, p. 450).

^33^Moody (2018, p. 39).

^34^Wright (2001, p. 109).

^35^Moody (2018, p. 39).

^36^See e.g., Khandelwal (2021), Jones (2022) and Bailey (2021a) “As the capabilities of blockchain expand, NFTs, or non-fungible tokens, have emerged as not only a revolutionary and innovative technology but one that is widely popular and ‘catchy' to the public;” but cf. Hector (2022).

^37^Bailey (2021b).

^38^Lester (2022).

^39^Cox (2021).

^40^https://v.cent.co/tweet/20 (featuring founder of Twitter, Jack Dorsey's, posting for sale his first-ever tweet “just setting up my twttr).”

^41^https://medium.com/swlh/how-to-sell-your-article-as-an-nft-a904690331fb (Khan, 2021).

^42^Roose (2021).

^43^https://www.theverge.com/2021/2/18/22287956/nyan-cat-crypto-art-foundation-nft-sale-chris-torres (Kastrenakes, 2021b).

^44^Garcia and Smith (2021).

^45^https://www.theverge.com/2021/3/1/22308075/grimes-nft-6-million-sales-nifty-gateway-warnymph (Kastrenakes, 2021a).

^46^Mendez (2021).

^47^Guadamuz (2021).

^48^Guadamuz (2021, p. 3).

^49^Guadamuz (2021, p. 4).

^50^Steiner (2022b, p. 4), a noted conceptual artist and IP lawyer, has offered a different metaphor for NFTs that readers still gaining familiarity with NFTs will likely find helpful, analogizing the NFT to a deed to a house with the house standing for what Steiner refers to as the Digital Resource (or asset that exists in creation with the token).

^51^Lewis et al. (2021).

^52^Appropriately referring to these as “Veblenesque bragging rights” (Steiner, 2022b, p. 4); see also Frye (2022) describing collectors who knowingly seek a form of “pwnership” rather than “ownership,” where the former refers to the right to exercise market clout and the latter refers to possessing exclusive rights of control.

^53^Pipkin (2021).

^54^“NFTs exist so that the crypto grifters can have a new kind of magic bean” (Gerard, 2021).

^55^Frye (2022, p. 346).

^56^Calling “digital rights management” “one of the most direct applications of the technology” (Kaczynski and Kominers, 2021).

^57^(“[T]he proof of your ownership is publicly available because it is stored on blockchain and it is decentralized. Basically, no one can steal it.”) (Lobanova, 2021).

^58^“That's why NFTs are important for consumers – they know y're buying the real thing” (Rennie, 2021).

^59^“While the Digital Age has created many new opportunities for artists, one of the biggest challenges has been around digital rights management and royalties. Digital files of any kind can be easily duplicated, so how does one discourage piracy while still leveraging the amazing global distribution network of the Internet? How does one make sure artists are able to reliably receive ongoing royalties for their work as digital versions of their content pass between buyers and sellers in both primary and secondary markets? And, how can you prove the digital artwork you own is “the real thing” vs. just a digital copy?” (Lester, 2022).

^60^“With blockchain, trust is established through mass collaboration and clever code in place of a powerful centralized institutions [*sic*] serving as middlemen” (Bailey, 2021b).

^61^Kaczynski and Kominers (2021).

^62^Frye (2022, p. 342).

^63^“NFTs have given graphic artists and content creators the chance to express themselves and earn money without getting involved with top galleries, auction houses, or other middlemen” (Frye, 2022).

^64^Despite claims of disintermediation, however, NFTs are minted on platforms by intermediaries who have come under fire for charging not insubstantial fees. See e.g,. Fadilpašić (2021) and Geron and the Fintech Team (2022).

^65^“[A]rtists have been deprived of capturing value by a sea of intermediaries like corporations and centralized platforms that have a monopoly on creative ownership. But now, NFTs (non-fungible tokens) are fundamentally altering many industries by creating a new paradigm for creative ownership directly between artists and their communities, detached from centralized companies” (Portion, 2021).

^66^Kaczynski and Kominers (2021).

^67^Identifying profile-picture NFT projects or “PFPs” as “the primary use case for images associated” with certain NFTs and stating that PFPs “are the reigning format for NFTs, both aesthetically and economically. Aesthetically, the format allows for experiments in variation within a standard template on a high-volume scale that Andy Warhol would have envied. Economically, it allows creators to supply a critical mass of semi-fungible intangible assets capable of sustaining an ecosystem of collecting, trading, market-making, and speculation” and calling Warhol “the progenitor of the PFP” (Steiner, 2022a, p. 1, 3).

^68^Fairfield (2022).

^69^Fairfield (2022, p. 9).

^70^Fairfield (2022, p. 9).

^71^Steiner (2022b, p. 17).

^72^Steiner (2022b, p. 5).

^73^Fairfield (2022, p. 12, 13).

^74^Reviewing possible theories of copyright infringement in connection with the minting of NFTs (Guadamuz, 2021, p. 19–20).

^75^Anonymous (2021), Steiner (2022b, p. 5).

^76^See e.g., Dafoe (2022), Donohue (2022), Rossow (2022), and Steiner (2022b, p. 5) (speculating that litigation might arise over the copyrightability of pixelated art such as a CryptoPunk, which Steiner, a proponent of experiments with cryptoart, believes to be the “reigning format for NFTs”).

^77^Hu (2022).

^78^Offering thoughtful expert analysis of the IP issues in a subset of the NFT market and using hypotheticals as well as his own minted NFT to illustrate the legal complexities (Steiner, 2022a, p. 7–13).

^79^Quoting Arry Yu, chair of the Washington Technology Industry Association Cascadia Blockchain Council (Conti and Schmidt, 2020).

^80^“The idea is that there is value in these items because they are unique” (Guadamuz, 2021, p. 27).

^81^“[T]his is what gives the NFT its ‘scarcity' value: it is supposed to be unique. In reality, anyone can mint as many versions of the same work as they wish” (Guadamuz, 2021, p. 6).

^82^Kaczynski and Kominers (2021), Steiner (2022b, p. 4.)

^83^Describing a “common misunderstanding” about what NFTs are and convey to purchasers (Steiner, 2022b, p. 4); Guadamuz, *supra* note 47, at 30 (stating that “the level of misinformation and misunderstanding of NFTs is staggering).

^84^Indeed, even industry accounts tend to conflate the token and the asset. See e.g., Financial News Media (2022) (“[T]he most common and lucrative application of NFTs in the sports industry will likely be the sale of limited edition video clips of sporting moments or player cards.”) (Guadamuz, 2021, p. 2, 9).

^85^n.12 (describing CryptoPunks as an example of NFTs whose digital assets are small enough to be natively on the blockchain) (Steiner, 2022a, p. 4).

^86^Fairfield (2022, p. 9).

^87^Guadamuz (2021, p. 8).

^88^Conti and Schmidt (2020).

^89^Locke (2022).

^90^Describing the current “crypto winter” but expressing optimism about the technologies (Shweigart, 2022).

^91^Portion (2021).

^92^Describing most NFTs as not natively stored on the blockchain (Steiner, 2022a, p. 4).

^93^Portion (2021).

^94^“most NFTs … merely link to metadata which in turn links to an image, either of which links may be severed, leaving the NFT owner with little more than an entry on a distributed ledger” (Steiner, 2022a, p. 4).

^95^Quoting Professor Robert Bocci of Georgetown University, an artist as well as a scholar, expressing caution about NFTs back in 2017: “Digital systems are extremely frail and vulnerable. An NFT link that works today may be broken tomorrow.” (Jones, 2022).

^96^“We now have a market where billions of dollars have been spent on NFTs and the majority of collectors lack the technical skill or an easy-to-use tool to assure the media files associated with their NFTs don't simply vanish.”

^97^Others have developed “forever storage” solutions that charge NFT collectors up-front for permanent storage of their image files (Bailey, 2021a).

^98^Hector (2022) stating that “[T]he environmental impact of cryptocurrency mining - a practice NFTs are reliant on - is huge” and collecting sources that estimate that the collective carbon footprint of just Bitcoin and Ethereum “would be the 48th worst CO2 polluter in the world; but given that over 8,000 currencies exist, the environmental impact is likely much worse”.

^99^Santos (2022).

^100^Calma (2021).

^101^Guadamuz (2021, p. 2).

^102^Bruner (2021).

^103^Gammon (2021).

^104^Pipkin (2021).

^105^Goodkind et al. (2020).

^106^Emmanuel (2021).

^107^“The Lightning Network is a layer-2 built on top of the Bitcoin network, meaning that it's built separately from the Bitcoin network but interacts with it. It's made up of a system of channels that allows people or companies to move money between one another without needing to use the blockchain to verify the transaction” (Hussey et al., 2016).

^108^One drawback to Flow and other such specialized blockchains is their underutilization: “Hardly anybody does NFTs on these chains — almost nobody uses them, and the local cryptocurrency for your fees is a lot more work to get hold of”.

^109^Noting that private blockchains “move away from what cryptocurrencies were supposed to do in the first place, which is create a decentralized network where anyone can make transactions without the oversight of a single institution.”

^110^Pipkin (2021).

^111^Dillet (2022).

^112^Jackson (2021).

^113^See e.g., Bruner (2021) and Calma (2021).

^114^Pipkin (2021).

^115^Beebe and Fromer (2018).

^116^Ouellette (2018).

^117^Said, Z.K. Collegiality Costs: Trademark Scarcity and Craft Beer's Politeness Problem, The Law and Economics of Trademark Law, Elgar Publishing, Glynn Lunney ed. (forthcoming, 2023).

^118^Leonard v. Stemtech International, Inc, 834 F.3d 376 (2016), (“Leonard III”).

^119^Leonard v. Stemtech Health Scis., Inc., No. CV 08-67-LPS, 2015 WL 4778827, at ^*^1 (D. Del. Aug. 13, 2015), aff'd sub nom. Leonard v. Stemtech Int'l Inc, 834 F.3d 376 (3d Cir. 2016).

^120^Leonard v. Stemtech Health Scis., Inc., No. CIV. A. 08-67-JJF, 2008 WL 5381359, at ^*^1 (D. Del. Dec. 19, 2008) (“Leonard I”). In 2012, Leonard filed a second action against various John Doe's associated with Stemtech's business and the actions were consolidated. Leonard v. Stemtech Health Scis., Inc., No. CV 08-67-LPS-CJB, 2013 WL 5288266, at ^*^1 (D. Del. Sept. 19, 2013).

^121^*Leonard III*, 834 F.3d at 381-382.

^122^*Leonard III*, 834 F.3d at 382.

^123^*Leonard III*, 834 F.3d at 383.

^124^Leonard v. Stemtech Health Scis., Inc., No. CIV.A. 08-067-LPS-CJ, 2011 WL 6046701, at ^*^4 (D. Del. Dec. 5, 2011), report and recommendation adopted, No. CA 08-067-LPS-CJB, 2012 WL 1133185 (D. Del. Mar. 28, 2012) [agreeing with defendants that “[p]laintiff cannot establish the necessary causal nexus between the generation of such profits and the infringement of Plaintiff's images required under 17 U.S.C. § 504(b)].

^125^Leonard v. Stemtech Health Scis., Inc., 981 F. Supp. 2d 273, 279 (D. Del. 2013) (“Leonard II”) (granting defendants' motion for summary judgment with respect to statutory damages).

^126^17 U.S.C. § 504(a).

^127^Yellowcake, Inc. v. Dashgo, Inc., No. 1:21-CV-0803 AWI BAM, 2022 U.S. Dist. LEXIS 10178, at ^*^8 (E.D. Cal. Jan. 19, 2022).

^128^Leonard III, 834 F.3d at 390 (internal citation omitted).

^129^Barrera and Burgos v. Brooklyn Music, Ltd., et al., 2004 U.S. Dist. LEXIS 12450 at ^*^8–9 (S.D.N.Y. June 30, 2004).

^130^On Davis v. The Gap, Inc., 246 F.3d 152, 165 (2d Cir. 2001).

^131^McRoberts Software, Inc. v. Media 100, Inc., 329 F.3d 557, 566 (7th Cir. 2003) [“The Copyright Act permits a copyright owner to recover actual damages suffered as a result of the infringing activity and any profits of the infringer resulting from the infringement that are not otherwise taken into account in calculating actual damages. 17 U.S.C. § 504(b)”]. The Act does not authorize recovery of punitive damages. *See* 17 U.S.C. § 504.

^132^Jarvis v. K2 Inc., 486 F.3d 526, 534 (9th Cir. 2007).

^133^The plaintiff's calculation of actual damages, based on its estimation of fair market value, started at $215,767.66 and was adjusted upwards via several multipliers discussed in detail below; defendants estimated actual damages, based on prior licensing fees, at $1,804. The parties' opposing positions explains some of the divergence of course, but the different methods of determining damages was also a factor.

^134^Leonard III, 834 F.3d at 385.

^135^Leonard III, 834 F.3d at 385. (citing the trial record).

^136^Leonard v. Stemtech Health Scis., Inc., No. CV 08-67-LPS, 2015 WL 4778827, at ^*^6 (D. Del. Aug. 13, 2015), aff'd sub nom. Leonard v. Stemtech Int'l Inc, 834 F.3d 376 (3d Cir. 2016) (“Stemtech was free to retain an expert who could have expressed this very opinion and calculated Leonard's actual damages based on his own licensing history—but that is not the trial strategy Stemtech chose to pursue”).

^137^Leonard v. Stemtech, Testimony of Plaintiff's Expert Witness, Jeffrey Brian Sedlik, 2013 WL 12122102 (D.Del.) (Expert Trial Transcript) (my emphasis).

^138^Sedlik's testimony emphasized this point in justifying his expansive valuation of the images: “Mr. Drapeau, who is the Chief Science Officer of Stemtech said that he [understood] … the value of Andrew's image, totally. It is a good representation. It is one of the early depictions anywhere. It is extremely valuable. … So I had a witness in the case and a representative of Stemtech both testifying that the image was rare and valuable.” *Id*.

^139^*Leonard III*, 834 F.3d at 389.

^140^Leonard v. Stemtech, Defendants Stemtech, Inc., et. al's Closing Statement and Plaintiff's Rebuttal 2013 WL 12123083 (D.Del.) (Trial Transcript) (“Dr. Rachel… a neurobiologist, and a Ph.D., … was drawn to become a distributor for the purpose of purchasing the product, and she was drawn to the product because of her review of articles that were published. She … was not drawn to either her distributorship or to the product because of any infringement or any Image 3 or Image 4”).

^141^Leonard v. Stemtech, Defendants Stemtech, Inc., et. al's Closing Statement and Plaintiff's Rebuttal 2013 WL 12123083 (D.Del.) (Trial Transcript).

^142^*Leonard III*, 834 F.3d at 390 (internal citation omitted).

^143^Mackie v. Rieser, 296 F.3d 909, 917 (9th Cir. 2002); accord *Jarvis v. K2 Inc*., 486 F.3d 526, 534 (9th Cir. 2007) (noting that fair market value is “an objective, not a subjective, analysis”).

^144^*Mackie*, 296 F.3d at 917 (my emphasis).

^145^Leonard v. Stemtech, Testimony of Plaintiff's Expert Witness, Jeffrey Brian Sedlik, 2013 WL 12122102 (D.Del.) (Expert Trial Transcript).

^146^McRoberts Software, Inc. v. Media 100, Inc., 329 F.3d 557, 566 (7th Cir. 2003).

^147^*See infra* notes 176-178 and accompanying text for further discussion.

^148^The Copyright Act does not provide for punitive damages. 17 U.S.C. § 504. *See e.g*. Grant Heilman Photography, Inc. v. McGraw-Hill Companies, 115 F. Supp. 3d 518, 526-527 (E.D. Pa. 2015); Faulkner v. Nat'l Geographic Soc., 576 F. Supp. 2d 609, 619 (S.D.N.Y. 2008) (“[T]he Copyright Act limits recovery in this case to “actual damages” and does not permit recovery of punitive damages. Whatever the industry may do or believe as a matter of voluntary and consensual practice does not trump Congress' limitation of damages for infringement in this case to actual damages”).

^149^Describing Congressional intent with the 1976 Act to create enhanced damages rooted largely in compensatory, not punitive principles and surveying cases that nonetheless suggest a punitive aspect in application (Samuelson and Wheatland, 2009); Examining and critiquing existing justifications for the supracompensatory aspect of statutory damages (Bracha and Syed, 2020).

^150^See 17 U.S.C. § 412 which limits the availability of statutory damages (1) to works infringed after the effective date of their registration or, (2) if published first and then registered, to works whose effective registration occurs no later than three months after publication.

^151^Faulkner, 576 F. Supp. 2d at 619.

^152^Leonard v. Stemtech Int'l Inc, 834 F.3d 376, 393 (3d Cir. 2016) (“The few district courts to consider the use of punitive multipliers have concluded that such use is improper under the Copyright Act… We agree with the reasoning of these district courts that, under the Copyright Act, an actual damages award may not include such a punitive component. We also agree with Leonard that this case does not involve the use of a multiplier to penalize unauthorized use. Rather, the record demonstrates that the multiplier here was used to calculate fair market value”).

^153^Straus v. DVC Worldwide, Inc., 484 F.Supp.2d 620 (S.D. Tex. 2007).

^154^In *Straus*, Sedlik testified on the impropriety of using multipliers to calculate fair market value. The district court cited Sedlik in its opinion: “In response to Straus's claim for a tenfold increase in actual damages, the defendants provide summary judgment evidence that a multiplier often times is not customary in the industry. Jeff Sedlik, defendants' damages expert, states /// that photographers may use licensing agreements that contain provisions for so-called retroactive licenses that include liquidated-damages provisions in the form of multipliers for any unlicensed use. … Such multipliers are a form of punitive damages. *If multipliers are included in a license agreement, they may apply as part of the parties' agreement, but would not otherwise bear on the fair market value of a license when infringement occurs*. Straus and DVC did not include a damage multiplier in their licensing agreement. According to Sedlik, absent such an agreement, using punitive multipliers to determine a fair market value of a license is “unreasonable and inappropriate.” *Straus*, 484 F.Supp.2d at 631-632 (emphasis added).

^155^Stemtech Int'l, Inc. v. Leonard, 138 S. Ct. 975 (2018).

^156^Leonard v. Stemtech Health Scis., Inc., No. CV 08-067-LPS-CJB, 2013 WL 5311295, at ^*^1 (D. Del. Sept. 23, 2013).

^157^Leonard v. Stemtech Health Scis., Inc., 981 F. Supp. 2d 273, 279–80 (D. Del. 2013).

^158^Leonard v. Stemtech Health Scis., Inc., 981 F. Supp. 2d 273, 279–80 (D. Del. 2013) (“It is worth noting that as to nearly all of the areas of testimony at issue, Professor Sedlik's expert reports do not contain record citations or otherwise make clear the factual bases underlying his statements. The Court can conclude that one of the statements at issue—that it is “ ‘likely' that Leonard has discovered only the proverbial “tip of the iceberg” of Stemtech infringements” —is not sufficiently linked to facts of record and should be stricken as unduly speculative. The statement, on its face, speculates about what is not known (and might or might not be the case) regarding alleged infringements”).

^159^Leonard v. Stemtech, Plaintiff Andrew Paul Leonard's Opening Statement, 2013 WL 12123072 (D.Del.) (Trial Transcript).

^160^Leonard v. Stemtech, Testimony of Plaintiff's Expert Witness, Jeffrey Brian Sedlik, 2013 WL 12122102 (D.Del.) (Expert Trial Transcript).

^161^17 U.S.C.102 (b).

^162^Leonard v. Stemtech Int'l, Inc., No. CIV.A. 12-86-LPS-CJB, 2012 WL 3655512, at ^*^1 (D. Del. Aug. 24, 2012), report and recommendation adopted, No. CIV.A. 12-86-LPS-CJB, 2012 WL 4591453 (D. Del. Sept. 28, 2012).

^163^Leonard v. Stemtech Health Scis., Inc., 981 F. Supp. 2d 273, 280 (D. Del. 2013) [“The portion of testimony that appears most likely based on a clear factual foundation, based on the nature of the testimony and that portion of Professor Sedlik's report, regards the scarcity of stem cell photographs in 2006 and how that fact impacts their value. (D.I. 202, ex. E; *id.*, ex. E−1 at 28–29)”].

^164^Leonard v. Stemtech Health Scis., Inc., No. CV 08-67-LPS, 2015 WL 4778827, at ^*^5 (D. Del. Aug. 13, 2015).

^165^Leonard v. Stemtech Health Scis., Inc., No. CV 08-067-LPS-CJB, 2014 WL 3367092, at ^*^1 (D. Del. July 8, 2014).

^166^Leonard v. Stemtech Health Scis., Inc., No. CV 08-67-LPS, 2015 WL 4778827, at ^*^2 (D. Del. Aug. 13, 2015).

^167^Grant Heilman Photography, Inc. v. The McGraw-Hill Companies, 115 F.Supp.3d 518, 526-27 (E.D. Penn. 2015) (“Although there is no United States Supreme Court or Third Circuit precedent on this issue, the majority of courts to have considered the issue have concluded that “actual damages” under the Copyright Act, 17 U.S.C. § 504(b), are limited to the fair market value of a license defendant would have obtained pre-infringement for use of the copyrighted work” and “actual damages” under copyright law must be limited to fair market value without added multipliers”).

^168^Leonard v. Stemtech Health Scis., Inc., No. CV 08-67-LPS, 2015 WL 4778827, at ^*^5 (D. Del. Aug. 13, 2015). (my emphasis).

^169^Leonard's images were “very popular,” in part because “for many, many years, they were the only ones [of their kind] that I could get and the only ones that I could make available to my clients.” … The images were also “beautiful.” Leonard v. Stemtech Health Scis., Inc., No. CV 08-67-LPS, 2015 WL 4778827, at ^*^6 (D. Del. Aug. 13, 2015) (memorandum order denying motions for new trial and remittitur).

^170^*Leonard III*, 834 F.3d at 389–390.

^171^*Leonard III*, 834 F.3d at 391.

^172^Stehrenberger v. R.J. Reynolds Tobacco Holdings, Inc., 335 F. Supp. 2d 466, 467 (S.D.N.Y. 2004).

^173^Hendlin et al. (2010).

^174^Stehrenberger v. R.J. Reynolds Tobacco Holdings, Inc., 2004 WL 3543361 (S.D.N.Y.) (Expert Testimony of Henri Dauman).

^175^“There is no windfall to the Plaintiff in the calculation of a fee with a formula use. … The Plaintiff is not calculating the highest use for which she might license the infringed image, but merely calculates the fee based on the use the Defendants made of the image. Based on that usage, the Plaintiff and I have arrived at a fair licensing fee and applied the multiplier about which the defendants complain.”

^176^Stehrenberger v. R.J. Reynolds Tobacco Holdings, Inc., 335 F. Supp. 2d 466, 467 (S.D.N.Y. 2004).

^177^Stehrenberger v. R.J. Reynolds Tobacco Holdings, Inc., 335 F. Supp. 2d 466, 467 (S.D.N.Y. 2004) at 468.

^178^Stehrenberger v. R.J. Reynolds Tobacco Holdings, Inc., 335 F. Supp. 2d 466, 467 (S.D.N.Y. 2004) at 469.

^179^Faulkner v. Nat'l Geographic Soc., 576 F. Supp. 2d 609 (S.D.N.Y. 2008).

^180^Faulkner v. Nat'l Geographic Soc., 576 F. Supp. 2d 609 (S.D.N.Y. 2008) at 613.

^181^Faulkner v. Nat'l Geographic Soc., 576 F. Supp. 2d 609 (S.D.N.Y. 2008) at 619.

^182^Faulkner v. Nat'l Geographic Soc., 576 F. Supp. 2d 609 (S.D.N.Y. 2008) at 619 (my emphasis).

^183^Evidence of intent is ordinarily relevant only when determining whether enhanced statutory damages may be appropriate. Faulkner v. Nat'l Geographic Soc., 576 F. Supp. 2d 609 (S.D.N.Y. 2008) at 613. (“The fact that the measure of actual damages here may well be a reasonable license fee, assuming a willing licensor and a willing licensee, is quite beside the point. In consequence, there is no proper role for proof of wilfulness. Its only function would be in service of an attempt by plaintiff to prejudice the jury's assessment of damages and, if it proves to be in issue, liability by portraying defendants in an unflattering light”).

^184^Grant Heilman Photography, Inc. v. McGraw-Hill Companies, 115 F. Supp. 3d 518, 526–27 (E.D. Pa. 2015) (citing cases).

^185^Grant Heilman Photography, Inc. v. McGraw-Hill Companies, 115 F. Supp. 3d 518, 526–27 (E.D. Pa. 2015) (citing cases). (“actual damages” do not include multipliers for unauthorized use, which courts have deemed impermissible penalties akin to punitive damages, which are not recoverable under § 504(b) of the Copyright Act”).

^186^Faulkner, 576 F. Supp. 2d at 617 (S.D.N.Y. 2008).

^187^Silberman v. Innovation Luggage, Inc., 2003 U.S. Dist. LEXIS 5420, at ^*^29-30 (S.D.N.Y. Mar. 31, 2003).

^188^Nimmer on Copyright § 14.02 (2022) (“It is suggested, therefore, that this rogue decision not be followed.”) The case in question is TVT Records v. Island Def Jam Music Group, 288 F. Supp. 2d 506, 509 n.5 (S.D.N.Y. 2003).

^189^Jerstad v. New York Vintners LLC, No. 18CV10470JGKOTW, 2019 WL 6769431, at ^*^3 (S.D.N.Y. Dec. 12, 2019), report and recommendation adopted, No. 18-CV-10470 (JGK), 2020 WL 58237 (S.D.N.Y. Jan. 6, 2020) (“Multipliers based on willfulness are only for consideration of statutory damages, not actual damages. *See* 17 U.S.C. § 504(c)(2)”). *See also* Faulkner, 576 F. Supp. 2d at 617 (collecting cases).

^190^Zahr K. Said, *Jury-Related Errors in Copyright*, _ Ind. L. J. (discussing the Jammie Thomas-Rasset litigation) (forthcoming 2022, on file with the author).

^191^In general, courts must respect damages awards “if there is a reasonable basis to do so.” Grant Heilman Photography, Inc., 115 F. Supp. 3d at 526–27 (E.D. Pa. 2015) (internal citation omitted); Leonard III, 834 F.3d at 392.

^192^Leonard III, 834 F.3d at 392.

^193^Leonard III, 834 F.3d at 393. (“These courts rejected the use of a multiplier or “ ‘fee for unauthorized usage' ” over and above what “would otherwise represent a fair and reasonable licensing fee for the infringed material” as a component of the actual damages calculation. *Stehrenberger*, 335 F.Supp.2d at 467. We agree with the reasoning of these district courts that, under the Copyright Act, an actual damages award may not include such a punitive component. We also agree with Leonard that this case does not involve the use of a multiplier to penalize unauthorized use. Rather, the record demonstrates that the multiplier here was used to calculate fair market value”).

^194^Feist Publications, Inc. v. Rural Tel. Serv. Co., 499 U.S. 340, 111 S. Ct. 1282, 1287, 113 L. Ed. 2d 358 (1991).

^195^Leonard v. Stemtech, Testimony of Plaintiff's Expert Witness, Jeffrey Brian Sedlik, 2013 WL 12122102 (D.Del.) (Expert Trial Transcript).

^196^Leonard v. Stemtech Health Scis., Inc., No. CV 08-67-LPS, 2015 WL 4778827, at ^*^5 (D. Del. Aug. 13, 2015).

^197^Leonard v. Stemtech, Testimony of Plaintiff's Expert Witness, Jeffrey Brian Sedlik, 2013 WL 12122102 (D.Del.) (Expert Trial Transcript).

^198^“Now, you won't see that in a licensing menu. In other words, you go to a stock agency website, it's not going to say rare, the level to which an image is rare.”

^199^Mannion v. Coors Brewing Co., 377 F. Supp. 2d 444, 452-54 (S.D.N.Y. 2005).

^200^Mannion v. Coors Brewing Co., 377 F. Supp. 2d 444, 452-54 (S.D.N.Y. 2005). at 453.

^201^Schulz (2017).

^202^See e.g., Time Incorporated v. Bernard Geis Associates, 293 F. Supp. 130, 145 (S.D.N.Y. 1968); Walsh v. Townsquare Media, Inc., No. 19-CV-4958 (VSB), 2022 WL 1302216, at ^*^2 (S.D.N.Y. May 2, 2022) (stating that while “a news reporting purpose by no means guarantees a finding of fair use,” … it is well established in this Circuit that “use of a copyrighted photograph” is generally fair “where ‘the copyrighted work is itself the subject of the story, transforming the function of the work in the new context”).

^203^Leonard v. Stemtech, Testimony of Plaintiff's Expert Witness, Jeffrey Brian Sedlik, 2013 WL 12122102 (D.Del.) (Expert Trial Transcript).

^204^Leonard v. Stemtech, Testimony of Plaintiff's Expert Witness, Jeffrey Brian Sedlik, 2013 WL 12122102 (D.Del.) (Expert Trial Transcript) (my emphasis).

^205^Stemtech Intern., Inc. v. Leonard, 2017 WL 382966 (U.S.) (petitioning based on the following question: “Does the Third Circuit's opinion, contrary to the precedent of the Federal, Second, Fourth and Fifth Circuits, improperly permit a plaintiff seeking actual damages under 17 U.S.C. § 504(a)(1) and (b) to disregard his pre-infringement licensing history and instead recover based upon the asking price of stock photography agencies for other authors' similar and dissimilar works without comparable uses that were never consummated licenses and then apply multipliers without a marketplace basis to arrive at an inflated damage award?”).

^206^Stemtech Int'l, Inc. v. Leonard, 138 S. Ct. 975 (2018).

^207^Search results accessed on Westlaw September 22, 2022.

^208^Affordable Aerial Photography, Inc. v. Palm Beach Real Est., Inc., 2021 WL 2823270, at ^*^4 (S.D. Fla. July 7, 2021) (noting that courts in the Eleventh Circuit “have declined to apply *Leonard*'s 7.4x multiplier to cases like ours, which don't involve microscopic photography” and collecting cases).

^209^Search results accessed on Westlaw May 30, 2022.

^210^For example, in Myeress v. Beautiful People Mag., Inc., No. 22-CV-20137, 2022 WL 1404596, at ^*^3 (S.D. Fla. May 4, 2022), the court fails to mention the exclusivity premium and characterizes the case this way: “*Leonard v. Stemtech Int'l, Inc*., 834 F.3d 376, 394 (3d Cir. 2016) (affirming a jury verdict of $1.6 million where the sum included three to five times the benchmark because of the scarcity factor”).

^211^Kjellberg et al. (2017), Vasiu and Vasiu (2020).

^212^See Rose (1998), at 135–136, Part III, at note 122 and accompanying text (discussing “proof of work,” NFTs and environmental costs).

^213^Rose (1998), at 135–136.
